# A phase II multi-institutional study assessing simultaneous in-field boost helical tomotherapy for 1-3 brain metastases

**DOI:** 10.1186/1748-717X-7-42

**Published:** 2012-03-21

**Authors:** George Rodrigues, Slav Yartsev, Keng Yeow Tay, Gregory R Pond, Frank Lagerwaard, Glenn Bauman

**Affiliations:** 1 Division of Radiation Oncology, Department of Oncology, University of Western Ontario and London Regional Cancer Program, London Health Sciences Centre, London, ON N6A4L6, Canada; 2 Department of Epidemiology and Biostatistics, University of Western Ontario, London, ON N6A3K7, Canada; 3 Department of Medical Biophysics, University of Western Ontario, London, ON N6A4L6, Canada; 4 Department of Medical Imaging, London Health Sciences Centre, London, ON N6A4L6, Canada; 5 Department of Oncology, McMaster University and Escarpment Cancer Research Institute, Hamilton, Ontario L8V1C3, Canada; 6 Department of Radiation Oncology, VU University Medical Center, de Boelelaan 1117, 1081, HV Amsterdam, The Netherlands; 7 A3-808, 790 Commissioners Rd. E, London, ON N6A4L6, Canada

**Keywords:** Brain metastases, Radiotherapy, Phase II, Clinical trial, Radiosurgery

## Abstract

**Background:**

Our research group has previously published a dosimetric planning study that demonstrated that a 60 Gy/10 fractions intralesional boost with whole-brain radiotherapy (WBRT) to 30 Gy/10 fractions was biologically equivalent with a stereotactic radiosurgery (SRS) boost of 18 Gy/1 fraction with 30 Gy/10 fractions WBRT. Helical tomotherapy (HT) was found to be dosimetrically equivalent to SRS in terms of target coverage and superior to SRS in terms of normal tissue tolerance. A phase I trial has been now completed at our institution with a total of 60 enrolled patients and 48 evaluable patients. The phase II dose has been determined to be the final phase I cohort dose of 60 Gy/10 fractions.

**Methods/Design:**

The objective of this clinical trial is to subject the final phase I cohort dose to a phase II assessment of the endpoints of overall survival, intracranial control (ICC) and intralesional control (ILC). We hypothesize HT would be considered unsuitable for further study if the median OS for patients treated with the HT SIB technique is degraded by 2 months, or the intracranial progression-free rates (ICC and ILC) are inferior by 10% or greater compared to the expected results with treatment by whole brain plus SRS as defined by the RTOG randomized trial. A sample size of 93 patients was calculated based on these parameters as well as the statistical assumptions of alpha = 0.025 and beta = 0.1 due to multiple statistical testing. Secondary assessments of toxicity, health-related quality-of-life, cognitive changes, and tumor response are also integrated into this research protocol.

**Discussion:**

To summarize, the purpose of this phase II trial is to assess this non-invasive alternative to SRS in terms of central nervous system (CNS) control when compared to SRS historical controls. A follow-up phase III trial may be required depending on the results of this trial in order to definitively assess non-inferiority/superiority of this approach. Ultimately, the purpose of this line of research is to provide patients with metastatic disease to the brain a shorter course, dose intense, non-invasive radiation treatment with equivalent or improved CNS control/survival and health-related quality-of-life/toxicity profile when compared to SRS radiotherapy.

**Trial registration:**

Clinicaltrials.gov - NCT01543542.

## Background

### Introduction

Historically, treatment for patients with metastatic disease in the brain has been palliative whole brain radiotherapy (WBRT) alone. Over the last 10 years, introduction of focal treatments (surgery or radiosurgery) for selected patients with metastatic disease has been explored both in clinical trials and institutional series. The treatment of patients with metastatic disease to brain now may include one or more of the following interventions: best supportive care, palliative WBRT, radiosurgery (SRS), or surgical resection with the goals of management being effective palliation of symptoms, preventing intracranial progression, preserving/stabilization of neurologic function, and maintenance of quality of life [1]. Typically, patients who are suitable for more aggressive, multi-modality therapies are those with oligometastatic disease (1-3 metastases), controlled extracranial disease, and good performance status. Randomized studies have demonstrated local control and neurologic progression free survival benefits for surgery or radiosurgery in conjunction with WBRT in this population when compared to WBRT alone, radiosurgery, or surgery alone [2,3].

### Stereotactic brain radiotherapy

Radiosurgery delivered as a single fraction to individual intracranial lesions has been established as a safe alternative to surgical resection for patients with oligometastatic disease within the brain [4-6]. The Radiation Therapy Oncology Group (RTOG) 9502 clinical trial established dose recommendations of 15-24 Gy for individual lesions up to 4 cm based on observed central nervous system toxicity at 3 months post treatment. This individualized approach has subsequently been shown to be safe and effective in numerous single institution and multi-institution reports and trials. For example, the multi-institutional randomized RTOG 9508 trial reported by Andrews et al., randomized 333 patients with 1-3 brain metastases between WBRT alone versus WBRT plus single fraction SRS boost [7]. Patients treated with WBRT and SRS were found to have improved/stable 6 month KPS and improved local control compared to WBRT alone. For the entire patient population a trend towards superior OS was observed in the WBRT and SRS cohort (6.5 vs. 5.7 months, *p *= 0.13) with a significant OS benefit for the subgroup of patients with single brain metastases (6.5 vs. 4.9 months, *p *= 0.04). The authors concluded that WBRT plus SRS should be considered standard treatment for patients with single brain metastases and considered for other patients with 2-3 brain metastases.

### Conceptual framework for fractionated SRS

Logistically, radiosurgery requires separate localization and treatment procedures that add some inconvenience and cost for patients, providers and caregivers. Depending on the radiosurgery system used, invasive immobilization devices may be necessary which can add to patient discomfort. Single fraction treatments also do not permit the exploitation of the potential radiobiologic benefits of reassortment and reoxygenation that may occur with a fractionated radiotherapy course. Hall et al. have argued that fractionated stereotactic radiotherapy may be more efficacious in the treatment of neoplastic disease compared to single fraction radiosurgery [8]. Additionally, tumor cell repopulation or sublethal damage repair may occur if there is a significant break between the radiosurgery and whole brain radiotherapy sessions. Finally, depending on the radiosurgery system used, treatment of more than 3 metastases may involve prohibitively long treatments, requiring multiple sessions or omission of radiosurgery entirely.

The introduction of in-room image guidance systems integrated with radiation treatment machines has led to the introduction of minimally invasive and non-invasive stereotactic radiosurgery/radiotherapy techniques. One such unit, Helical tomotherapy (HT) combines intensity modulated fan-beam radiotherapy delivery with megavoltage computed tomography (MVCT) imaging for integrated patient positioning and treatment delivery [9,10]. Such a combination provides a potential alternative to conventional stereotactic frame systems for precision radiotherapy [11]. Dosimetric evaluation of HT delivery for primary and metastatic brain tumors have suggested similar normal tissue sparing and target coverage compared to other precision radiotherapy techniques. HT (as well as other forms of intensity modulated radiotherapy delivery) lends itself to synchronous boost strategies as multiple targets can be easily treated to different dose (and dose per fraction) levels in the course of the intensity modulated radiation delivery [12].

Therefore, HT could potentially allow for radiosurgery-type boost treatments to be given synchronously with the standard whole brain radiotherapy dose and in this way be used to efficiently boost multiple brain metastases without the need for separate stereotactic procedures. From a dosimetric standpoint, the ability to incorporate boost contributions with larger field volumes as part of the treatment planning optimization process provides an advantage of the simultaneous boost strategy over sequential whole brain radiotherapy with radiosurgery boost. This advantage occurs as the radiosurgery boost dose is added to the previously delivered whole brain dose without opportunity for optimization of these two components potentially resulting in unintended increased dose to the brain. In the case of the treatment of brain metastases using the SIB technique the lower isodose "spill" from the SIB can be incorporated as a component contributing to the whole brain radiation dose allowing the simultaneous optimization of both components and improved dose distribution compared to sequential WBRT and SRS boosts.

In a discussion of linear accelerator delivery techniques using fixed circular collimators, Podgorsak et al. noted improved conformity for non-coplanar delivery compared to single plane rotational delivery suggesting coplanar arc approaches should be at a disadvantage compared to conventional stereotactic non-coplanar techniques [13]. In HT fan beam arc therapy; however, the ability to modulate the beam intensity from multiple (51 per rotation and multiple overlapping rotations in the case of HT) coplanar projections helps offset the disadvantage of standard coplanar delivery. In comparisons of serial tomotherapy to gamma knife delivery [14] and HT to conventional photon and proton stereotactic techniques [15,16], fan beam arc therapy has been demonstrated to provide comparable conformity with excellent target dose homogeneity. Thus, tomotherapy would appear to be a feasible alternative for precision radiotherapy to small intracranial volumes.

Clearly, increased intracranial control can carry benefits to the patient in terms of a decreased rate of neurologic morbidity and death from progressive intracranial disease. However, in the setting of metastatic cancer, expected survival is limited and in the randomized trials reported, an overall survival benefit from SRS + WBRT or surgery + WBRT has not uniformly been noted. From a societal point of view, it may be difficult to justify resource intense treatments such as craniotomy or radiosurgery. Additionally from a patient point of view, it may be difficult to justify the inconvenience and side effects of these treatments in the face of a limited survival benefit. Improvements in technology to achieve improved intracranial disease control while minimizing resource use and patient inconvenience are needed for this common cancer problem. Thus, the combination of WBRT with a synchronous boost may carry significant therapeutic gain for the patient with brain metastases compared to the conventional combination of WBRT with a separate SRS boost.

### Preclinical investigations/considerations

In order to calculate potentially equivalent biological doses for fractionated radiotherapy when compared to SRS techniques, the linear quadratic (LQ) equation is frequently utilized for these calculations. This equation provides a method of comparing different radiation fractionation schemes according to their Biologically Effective Dose (BED):

BED=nd*(1+d/(α/β))

where n = number of fractions; d = dose/fraction; α/β = 10 for tumour effects; 3 for late effects

The LQ equation is widely accepted as a useful tool for comparing fractionation schemes and clinical trials of altered fractionation based on predictions by the model, and have confirmed its accuracy in predicting late effects for different radiation schedules. For the purposes of this study the tissue tolerances based on the BED equivalent tissue derived from available phase I/II and II trials of radiosurgery added to whole brain radiotherapy for patients with brain metastases are used for comparison. Within the RTOG 9005 radiosurgery dose escalation protocol, metastasis in the 2-3 cm range were treated with a radiosurgery dose of 18 Gy, while smaller lesions were treated with doses up to 24 Gy without toxicity [17]. Within the RTOG 9508 trial, radiosurgery boosts of 15-20 Gy were used in addition to a WBRT of 37.5 Gy in 15 fractions for a total lesional BED dose of 159-222 Gy. Using LQ equation we calculated that with the simultaneous infield boost technique, an intra-lesion dose of 45- 60 Gy/10 fractions would provide a similar intra-lesion BED range to that delivered within the RTOG 9508 from the combination of whole brain radiotherapy plus radiosurgery.

We modelled the feasibility of a simultaneous in field boost (SIB) to individual brain metastases during a course of whole brain radiotherapy using helical tomotherapy IMRT [18]. Planning CT data from 14 patients with 1-3 brain metastases treated with conventional palliative whole brain radiotherapy were used to model an intralesional SIB delivery that provided a total intralesional dose of 60 Gy with a surrounding whole brain dose of 30 Gy (designed to be isoeffective to whole brain radiotherapy of 30 Gy with an 18 Gy/1 fraction radiosurgery boost). Accuracy of treatment on the helical tomotherapy unit was verified in a phantom using radiographic films and point dose measurements versus calculated planning data. In all cases, SIB to 60 Gy with WBRT to 30 Gy was possible while maintaining critical structures below assigned dose limits. Estimated radiation delivery time for the SIB treatment was approximately 10 minutes per fraction. Planning and treatment of the head phantom was associated with an overall accuracy of 2 mm. Comparison to conventional non-co-planar arc fractionated stereotactic radiotherapy plan demonstrated similar target coverage and improved critical tissue sparing even for a challenging anatomy with multiple lesions in the same plane as the optic apparatus. Based on this modelling study, use of an image guided SIB on HT seemed potentially feasible and a phase I trial was initiated.

### Phase I clinical trial results

Based on the preclinical dosimetric results, a phase I clinical trial was initiated in 2006 to explore the HT SIB brain metastases concept at three institutions (London, Ontario (lead institution), Ottawa, Ontario and Madison, Wisconsin) [19]. The study objective was to determine the maximally tolerated dose (MTD) of synchronous in-field boost (SIB) HT RT integrated with WBRT for the palliative treatment of patients with 1-3 brain metastases. The phase I trial was approved by the Institutional Review Boards at the participating institution and registered (Ontario Cancer Clinical Trials Registry OCT 1145 TOMO-B) as per CONSORT guidelines. Patient eligibility for the trial was as follows: histologically proven cancer, imaging and clinical presentation consistent with brain metastases; 1-3 brain metastases on pretreatment contrast enhanced CT or MRI, lesion size ≥ 5 mm and ≤ 3 cm in diameter, lesion > 5 mm from brainstem optic or optic apparatus, Karnofsky Performance Status ≥ 70, extracranial disease absent, controlled or planned to be treated (in the case of synchronous presentation), and anticipated survival greater than 3 months. The trial was designed according to typical phase I dose escalation rules with 5 dose levels for the SIB boost: 35, 45, 50, 55, and 60 Gy. The trial was designed to accrue 3 patients at each dose level with a subsequent escalation if no dose limiting toxicity (DLT) was seen at 3 months with a further 3 patients to be enrolled if one patient experienced DLT. During the three-month waiting period for the dose level under assessment we allowed enrolment at the previously evaluated dose level one step below the current dose level.

Toxicity was monitored weekly during treatment and every month for 3 months post treatment then every 3 months for one year. Response on imaging at 12 weeks post treatment was assessed. Patients were accrued at 3-6 patients per dose level and escalation to the next dose level occurs if no limiting (grade 3 or greater) toxicity was observed in more than 1 of 3 or 2 of 6 patients by three months post treatment. This endpoint was designed to be similar to the RTOG 9005 radiosurgery dose finding study. Patients were also monitored for long-term toxicity, understanding that the treatment paradigm being explored is novel and that important CNS toxicity endpoints, such as radionecrosis might manifest after the initial three-month observation point.

A total of sixty patients were registered for potential treatment in the phase I trial. Twelve patients (20%) were excluded from the analysis, due to treatment refusal (n = 6), ineligibility to receive treatment due to decline in performance status related to progressive disease (n = 4), or subsequent loss to follow-up after treatment (n = 2). Therefore, a total of 48 (80%) treated patients were included for the DLT analysis. The mean age of patients on protocol was 65.0 years (range 39-90 years). Thirty (63%) patients were male and median Karnofsky performance status was 80 (range 70-100). A total of 24 (50%) of patients were diagnosed with a lung primary (23 non-small cell lung cancer, 1 small cell lung cancer). A total of 70 lesions were treated with 3 patients having 3 lesions, 16 patients with 2 lesions, and 29 patients with solitary lesions. Median lesion size was 1.38 cm (range 0.5-3 cm).

A total of 32/48 (66%) of cases were evaluable at three months post treatment with imaging; 29/48 (60%) had their planned 3 month clinical visit as well. Among the 16 (33%) cases that did not have imaging at 3 months, reasons for non-evaluation were progressive extracranial disease (n = 14) or early intracranial recurrence (n = 2). Of those patients with imaging but no clinical follow-up at 3 months (n = 3), reasons for no clinical follow-up included two patients with documented extracranial progression and one patient with combined intracranial/extracranial progression based on reports from local providers. In terms of dose level and availability for evaluation, all three level one (35 Gy) cases were evaluable by imaging at three months. Five of 16 (31%) level two (40 Gy) cases, 4/15 (27%) level three cases (50 Gy), 4/8 (50%) level four (55 Gy) cases and 1/6 (17%) level five (60 Gy) were found to be non-evaluable by imaging at three months. No statistical association between dose level and non-evaluation rate was found to exist in this patient population (chi square *p *= 0.60). That is, a similar proportion of patients were non-evaluable at each dose level, decreasing the probability that there was an underestimation of dose limiting toxicity due to a difference in early patient attrition at the dose levels examined. There were 2/4 patients treated at the highest dose level (level five, total of 60 Gy lesion dose) who were not evaluable at 3 months. One patient did have a follow-up MRI scan prior to the three-month mark, which demonstrated progressive CNS disease that accounted for his death prior to the three-month clinical follow-up. The other patient had documented progressive extracranial metastatic disease prior to death with no neurologic signs or symptoms to suggest treatment related toxicity.

No cases of grade III-V DLT were found to possibly, probably, or definitely be attributable to protocol treatment in any of the study cohort levels examined in the Phase I study. Median survival of all patients was 5.29 months (range 0.49-31.2 months). Seven patients were still alive at the time data analysis (09/09). Median follow-up of all living patients was 7.72 months (range 3.4-24.2 months). No treatment related late toxicity was noted among this subgroup of patients. Of the 32 patients radiologically assessed at the three month period of time; 2 patients experienced a complete response, 16 a partial response, 6 demonstrated stable disease, and 8 had progressive disease, for a crude rate of stable or responding disease of 75% (24/32). Of the 8 patients with progression, 4 patients had local progression in the SIB treated lesions, 2 patients had intracranial but non-local CNS progression (i.e. outside of the SIB treated lesions), and another 2 patients had both local and CNS progression on 3 month imaging.

In summary, we were able to achieve our target dose of 30 Gy WBRT with a simultaneous boost of individual lesions up to 60 Gy, both delivered over 10 fractions. No dose limiting toxicity at the three-month assessment was noted at any of the dose levels and among the subset of patients living beyond 3 months no treatment related late toxicity was noted. A number of patients enrolled on our trial missed complete follow-up (due to lack of imaging or clinical assessment) at the three-month point exclusively due to extracranial, intracranial progression or intercurrent disease. In all cases, it was determined that this early deterioration was not due to treatment related toxicities. In terms of patient outcome, partial responses were seen at all dose levels, without a clear increase in response rate with increasing SIB dose due to the limited numbers included in this phase I study. The three-month non-evaluable rate in our phase I clinical study is consistent with the RTOG 9508 post-treatment dropout rates. In addition, formal neurocognitive testing or quality of life assessments were not included in this study thus the impact of the SIB approach in these domains remains to be demonstrated.

### Formulation of phase II clinical trial

Ideally, a phase III equivalence study would be desirable to confirm whether SIB-HT treatment as developed in our Phase I trial is equivalent to WBRT + SRT in terms of patient outcomes. However, a phase III equivalence study would require a prohibitively large sample size, which is not justifiable based on present information. Thus, we propose that a single-arm phase II study is warranted to ensure that efficacy outcomes with HT are not significantly worse than what would be expected for patients given WBRT + SRS. HT would be considered of interest for study in a phase III equivalence trial compared with WBRT + SRS if preliminary evidence indicated that HT was not substantially inferior to WBRT + SRS. However, if a phase II study indicated that efficacy outcomes for patients given HT were significantly worse than WBRT + SRS, than performing a phase III equivalence study would not be justifiable. Thus, this phase II study will explore whether outcomes for patients treated with HT are significantly worse than outcomes for patients treated with WBRT + SRS.

## Methods/Design

### Study objectives/hypotheses

The primary objective of interest in this study is to obtain an estimate of median overall survival (OS), six-month intracranial control (ICC) and six-month intralesional control (ILC), in order to ensure that the fractionated treatment is not substantially inferior to RTOG historical controls. Secondary objectives include longitudinal RECIST criteria response rates, cognitive assessment, health-related quality-of-life, and treatment-related toxicity.

The study null hypothesis is that fractionated HT SIB RT is not inferior to reported RTOG 9508 SRS outcomes of OS, ICC, and ILC. The study alternative hypothesis is that fractionated HT SIB RT is inferior to ANY reported historical RTOG SRS outcomes of OS, ICC, and ILC.

### Study design overview

The proposed clinical trial is designed to be a phase II multi-institutional assessment of helical tomotherapy simultaneous in-field boost radiation to 1-3 brain metastases in patients with a variety of primary malignancies. A common dose of the 60 Gy MTD in 10 fractions will be used from the result of the preceding phase I dose-escalation trial as coordinated by the London Regional Cancer Program. No blinding or randomization procedures are required in this protocol. However, maximal accrual to the following four bins will be respected in order to balance overall patient population with respect to the primary site of disease as well as brain metastasis number for an accurate historical comparison to the RTOG stereotactic trial.

Bin #1: Lung Primary and Solitary Metastasis, n = 33

Bin #2: Lung Primary and 2-3 Metastasis, n = 19

Bin #3: Non-Lung Primary and Solitary Metastasis, n = 26

Bin #4: Non-Lung Primary and 2-3 Metastasis, n = 15

Inclusion criteria for this research protocol will include: a histologic diagnosis of primary cancer, a contrast-enhanced MRI demonstrating 1-3 metastases within 6 weeks of study enrollment, patient age ≥ 18 years, KPS ≥ 70, patient to be available for subsequent follow-up appointments and diagnostic testing, anticipated survival (independent of the brain metastases) > 3 months, patient informed consent obtained, and the extracranial disease must be controlled, to be treated, or absent. Exclusion criteria for this protocol will include: metastases not suitable for synchronous boost (> 3 lesions or any lesion size > 3 cm in maximum dimension, metastases close to (within 5 mm) brainstem or optic apparatus, cytologic or imaging evidence of leptomeningeal spread, intracranial extension of an osseous (calvarial) metastasis, or evidence of intraventricular or subependymal growth), no prior histologic confirmation of malignancy, underlying medical condition precluding adequate follow-up, prior cranial radiotherapy, concurrent cytotoxic chemotherapy, lack of informed consent, patient unable to complete study questionnaires, and any contraindications to MRI or gadolinium contrast.

### Study endpoints and sample size calculation

The primary outcome measure of this study has been selected after review of the literature regarding recent (RTOG 9508 and RTOG 0614) clinical trials assessing radiotherapy for brain metastases. The RTOG trials have focused on overall survival and intracranial control of disease as endpoints of interest. For example, patients treated with WBRT + SRS on the RTOG 9508 trial experienced median overall survival of approximately 6.5 months with 6-month intracranial tumour progression-free rate of approximately 85% and 6-month local (lesion) control rate of approximately 90%.

We hypothesize HT would be considered unsuitable for further study if the median OS for patients treated with the HT SIB technique is degraded by 2 months, or the intracranial progression-free rates (ICC and ILC) are inferior by 10% or greater compared to the expected results with treatment by WBRT + SRS. In other words if in our phase II trial OS ≤ 4.5 months, 6-month intracranial progression-free (ICC) rate is ≤ 75%, OR 6-month local control (ICL) rate is ≤ 80%, HT SIB RT would be considered to be inferior to the RTOG historical control data for WBRT+SRS and should not proceed to phase III testing. To account for multiple testing, each statistical assessment of each individual outcome will be set at the α = 0.025 and β = 0.10 levels. All tests will be 1-sided and calculations will be performed using the on-line calculators available at (http://www.crab.org/Calculators.asp - Southwest Oncology Group (SWOG) Cancer Research and Biostatistics (CRaB) department). It is assumed that for this study, patients will be accrued over two years and there will be 6 months of follow-up after the last patient is accrued. Therefore, for overall survival, set H0: median OS = 6.5 months versus HA: median OS = 4.5 months, 93 total patients are required. For the ICC progression-free rate, set H0: 6-month rate = 85% versus HA: 6-month rate = 75%, 71 total patients are required. For ILC progression-free rate, set H0: 6-month rate = 90% versus HA: 6-month rate = 80%, 53 total patients are required. Given the actuarial nature of the six-month OS endpoint, no additional adjustment for loss to follow-up is required. Thus, we will aim to accrue a total of 93 enrolled and on-treatment patients, as this will be sufficient to assess all outcomes of interest (OS, ICC, ILC).

### Logistical issues

Given the multi-institutional nature of this clinical trial, several pre-requisites exist for centres to participate in this clinical trial. Centres must have Helical Tomotherapy technology available at their centre in order to participate in this clinical trial. A copy of local REB and local clinical trial unit approval letters must be sent to coordinating centre PI prior to initiation of this study. Participating centres must submit a three metastases test case planned with HT with phantom delivery and measurement data to demonstrate the centre's ability to deliver protocol treatment. This three-metastasis test case will be provided to the participating sites by the London investigators in order to provide a relative quality assurance assessment of the various participating centres. This planning and phantom data will require quality assurance approval by physics PI prior to clinical trial initiation and patient enrolment. In conjunction with this physics submission, all default planning importance, penalty factors, and precedence factors to be submitted to and approved by coordinating centre PI. Any required modifications to the protocol, letter of information, consent form, and/or local case report forms need to be approved by coordinating centre prior to patient enrolment to ensure compatibility with the central study database.

### Study procedures

All patients must have a contrast enhanced MRI brain within six weeks of study enrolment, a dictated history including steroid and anti-convulsant usage, dictated physical examination, screening neurological examination with mini-mental status examination (MMSE), FACT-Br HRQoL questionnaire completed during enrolment visit, and a baseline NCI-CTC V3 toxicity assessment. All medications received by the study subjects prior to initiation will be recorded on the subject's case report form. Specific treatment simulation and planning details with relation to the delivery of 60 Gy SIB with 30 Gy WBRT are available in the attached clinical protocol (see Additional file 1). Steroid, anticonvulsant use and cancer therapies/medications delivered after treatment initiation will be recorded. Cytotoxic chemotherapy is not permitted during the radiotherapy. Patients may be treated with glucocorticoids and prophylactic anticonvulsants as required.

Follow-up visits and assessments (KPS, NCI CTC V3 Toxicity, FACT-Br HRQoL, steroid, and anticonvulsant medications) will occur at the following intervals post treatment: 6 weeks and 3, 6, 9, 12, 18, and 24 months. Contrast enhanced MRI brain scans will occur at 3 and 6 months after radiation therapy completion. Other CT or MRI based imaging will occur thereafter according to institutional standard of care guidelines prior to subsequent visits. Participating centers will be required to declare their standard of care imaging past 6 months to the study principal investigator prior to patient enrolment.

### Statistical analysis plan

Descriptive statistics will be used to summarize the patient population, tumor- and treatment-related parameters, treatment toxicities, causes of death, location of intracranial, intralesional and extracranial progression and performance status. Time to event outcomes will be estimated using the Kaplan-Meier method. Tables and plots will be used to better illustrate results. 95% confidence intervals will be constructed for outcomes of interest. A calculation of actuarial six-month OS, ICC, and ILC will be performed after all patients have had 6 months of potential follow-up in order to assess the phase II research question to either accept or reject the study hypothesis that HT SIB RT is a clinically appropriate alternative to WBRT+SRS.

## Discussion

### Knowledge dissemination plan

Successful completion of this phase II clinical trial will allow for a publication regarding the clinically significant outcomes, related to fractionated HT-based radiosurgery, such as overall survival, intracranial control, and local intralesional control. Venues for publication include the various primary radiation oncology journals and presentations can occur at national oncology meetings such as the Canadian Association Radiation Oncologists, American Society of Therapeutic Radiation Oncology, and the American Society of Clinical Oncology. Presentations at other meetings including brain and supportive care specific conferences can further disseminate any scientific information gained from this research program. Subsequent presentations to cooperative groups (e.g. RTOG, NCIC) with respect to a formal phase III study will depend on the results reported with respect to this study.

### Potential study obstacles

The main obstacle that potentially may interfere with successful completion of this trial is low patient accrual. Ongoing monitoring of accrual will be performed in order to continually assess this issue. Accrual updates will be performed to all participating institutions in order to maximize the exposure of this study. Further review of inclusion/exclusion criteria may be necessary, in future, to ensure that barriers to appropriate enrollment of patients are minimized.

### Study timelines

November 2009: OICR Grant Application Complete

January 2010: REB Approval at LHSC Obtained (UWO REB 16776)

Spring 2010: OICR Research Funding Confirmed (09NOV-259)

Spring 2010: Initiation of Clinical Protocol at LHSC site

July 2010: REB Approval and Protocol Initiation at Other Participating Institutions

June 2013: Expected Completion of Accrual

January 2014: Six-month Follow-up Completed*

February 2014: Data analysis of Primary Endpoint Completed*

Spring 2014: Abstracts Completed for National Meetings*

Fall 2014: Research Presentations, Preparation of Manuscripts on Phase II Study*

*Depending on actual date of accrual completion.

### Investigator team

The principal investigator of this project will be ultimately responsible for the conduct of the clinical trial. GR has been awarded a clinician scientist designation through the Ministry of Health Ontario-Ontario Association of Radiation Oncologists program that allows for dedicated research time to ensure the proper supervision of this research to its completion. GB (Radiation Oncologist, coinvestigator) has extensive clinical and research experience with helical tomotherapy and CNS tumours. SY is a coinvestigator in the area of medical physics and helical tomotherapy who will provide physics support for the dosimetric and clinical trial components of this research program. KT (Neuroradiologist) will provide support for interpretation of CNS response/relapse determination.

### Participating sites

Participating site collaborators from Ottawa, Montreal-CHUM, Montreal-McGill, and Edmonton will coordinate the activation of this clinical trial at all their respective TomoTherapy centres. This clinical trial will, therefore, be the broadest Canada-wide HT collaboration since clinical implementation of HT in 2003. In addition, ongoing external collaborations with non-participating sites (primarily Dr. F Lagerwaard - VU Amsterdam) will augment our capability for innovative hypothesis generation with relation to oligometastatic brain disease (1-3 vs. > 3 mets), fractionation selection (5 versus 10 fractions), and radiation platform selection (Tomotherapy^® ^versus RapidArc^®^) as well as other hypotheses. Although this institution is not formally part of the phase II protocol, this external collaboration may assist in the eventual design, implementation, and successful completion of a future definitive phase III clinical trial [20].

## Abbreviations

BED: Biologically effective dose; Br: Brain; C: Cervical; CNS: Central nervous system; CONSORT: Consolidated standards of reporting trials; CT: Computed tomotherapy; CTC: Common toxicity criteria; DLT: Dose limiting toxicity; FACT: Functional assessment of cancer therapy; GTV: Gross tumor volume; Gy: Gray; HRQoL: Health-related quality-of-life; H0: Null hypothesis; HA: Alternative hypothesis; HT: Helical tomotherapy; ICC: Intracranial control; ILC: Intralesional control; IMRT: Intensity modulated radiation therapy; KPS: Karnofsky performance status; L: Left; LQE: Linear quadratic equation; MMSE: Mini-mental status examination; MRI: Magnetic resonance imaging; MTD: Maximum tolerated dose; NCI: National cancer institute; OS: Overall survival; PI: Principal investigator; R: Right; REB: Research ethics board; RECIST: Response evaluation criteria in solid tumors; RTOG: Radiation therapy oncology group; SIB: Simultaneous infield boost; SRS: Stereotactic radiosurgery; Sx: Surgery; WBRT: Whole brain radiation therapy.

## Competing interests

LRCP has been awarded a TomoTherapy Research Partnership Grant (PI George Rodrigues) for partial funding of this phase II clinical trial. The VUmc Amsterdam has a research agreement with Varian medical systems and Brainlab AG.

## Authors' contributions

GR is the principal investigator and principal author of the clinical protocol/research grant. SY is the primary medical physics lead/author for the clinical protocol/research. KT is the primary radiological imaging lead/author for the clinical protocol/research grant. GP is a statistical consultant that led the sample size calculation for the clinical protocol/research grant. FL is a primary scientific research collaborator with regards to brain metastases simultaneous in-field radiation therapy between LHSC and VUmc. GB is the co-principal investigator and author of the clinical protocol/research grant. All coauthors have reviewed the contents of this research protocol and manuscript.

## Supplementary Material

Additional file 1 **Clinical Trial Protocol**. Click here for file
