# Supplementary material for: A phase II multi-institutional study assessing simultaneous in-field boost helical tomotherapy for 1-3 brain metastases
Source: Radiat Oncol. 2012 Mar 21;7:42. doi: 10.1186/1748-717X-7-42 (PMC3341183; doi:10.1186/1748-717X-7-42)
Supplement: Additional file 1 — Clinical Trial Protocol. [file 1748-717X-7-42-S1.PDF]

Clinical Trial Protocol

A Phase II Multi-institutional Study Assessing Simultaneous In-field Boost Helical Tomotherapy for 1-3 Brain Metastases

Nominated Principal Investigator:
George Rodrigues, MD, FRCPC, MSc
Clinician Scientist, London Regional Cancer Program (LRCP)
Associate Professor, University of Western Ontario (UWO)
A3-808 790 Commissioners Rd E
London, Ontario N6A 4L6
Phone: 519 685 8500 ext 52833
Fax: 519 685 8736
Email george.rodrigues@lhsc.on.ca

Principal Investigator (London):
Dr. Glenn Bauman
Chair and Professor of Oncology, LRCP and UWO
790 Commissioners Rd E
London, Ontario N6A 4L6
Phone: 519 685 8500 ext 53177
Fax: 519 685 8736
Email glenn.bauman@lhsc.on.ca

Other Principal Investigators:

Dr. Keng Yeow Tay (Imaging, LHSC/UWO)
Dr. Slav Yartsev (Physics, LRCP/UWO)

INDEX

Schema....3

Eligibility Checklist....4

1.0 Introduction....5

2.0 Objectives....8

3.0 Patient Selection....9

4.0 Pre-treatment Evaluations and Management....11

5.0 Study Logistics....12

6.0 Trial Design....13

7.0 Radiation Therapy....15

8.0 Patient Assessments....20

9.0 Adverse Events....22

10.0 Subject Discontinuation/Withdrawal....26

11.0 Data Collection....27

12.0 Statistical Considerations....30

13.0 Publication Policy....31

References

Appendix I	Letter of Information and Consent Form
Appendix II	Performance Status Scoring
Appendix III	Sample SIB Helical Tomotherapy Plan
Appendix IV	RECIST Criteria
Appendix V	FACT-Br HRQoL Questionnaire
Appendix VI	Mini-Mental Status Examination (MMSE)

SCHEMA

ENROLLMENT

**Record Baseline Patient, Tumour, and Treatment Factors,
FACT-Br Health-Related Quality-of-Life, MMSE, and NCI-CTC toxicity**

↓

TREATMENT

**Whole Brain XRT 30Gy/10 fractions with
Simultaneous Infield Boost of Brain Lesions to 60 Gy**

↓

FOLLOW-UP

**Primary Outcomes: Overall Survival, Local and CNS Control (RTOG criteria)
Secondary Outcomes: RECIST Criteria Response, NCI-CTC Toxicity, MMSE, and
FACT-Br Health-Related Quality-of-Life**

Patient Population:

- Histologic diagnosis of primary cancer.
- Contrast enhanced MRI
- Age ≥ 18
- KPS ≥ 70
- Patient available for subsequent follow-up appointments
- Anticipated survival > 3 months
- No previous brain radiation or concurrent chemotherapy
- No contraindications to high dose brain radiation therapy
- Extracranial disease controlled or to be treated
- No contraindications to contrast enhanced MRI

Required Sample Size: 93 Patients

ELIGIBILITY CHECKLIST

1. Histologic diagnosis of primary cancer (**Yes** / No)

2. Contrast enhanced MRI demonstrating 1-3 metastases within 6 weeks of enrollment (**Yes** / No)

3. Age ≥ 18 (**Yes** / No)

4. KPS ≥ 70 (**Yes** / No)

5. Patient available for subsequent follow-up appointments and testing as well as health-related quality of life questionnaires (**Yes** / No)

6. Anticipated survival (independent of the brain metastases) > 3 months (**Yes** / No)

7. Patient informed consent obtained (**Yes** / No)

8. Metastatic suitable for synchronous boost (**Yes** / No)

9. Underlying medical condition precluding adequate follow-up (**Yes** / No)

10. Prior cranial radiotherapy (Yes / **No**)

11. Concurrent cytotoxic chemotherapy (Yes / **No**)

12. Extracranial disease controlled or to be treated (**Yes** / No)

1.0 Introduction

Brain metastases are a common cancer problem and the results with current treatments are generally unsatisfactory. The majority of patients with brain metastases are treated with a 1-2 week course (20-30Gy) of whole brain external beam radiotherapy (WBXRT) with an expectation of improvement in survival from 1-2 months with supportive care alone to 4-6 months. Selected subgroups of patients (younger age, good performance status, extra-cranial metastases absent or controlled) achieve longer survivals with standard external beam radiotherapy. One group of patients with a better prognosis is those presenting with solitary brain metastases (single brain metastasis with no other active systemic cancer) and these patients may benefit from aggressive local treatment of their metastases with surgery followed by whole brain radiotherapy.

Stereotactic radiosurgery (SRS) is emerging as a less invasive, equally efficacious alternative to surgical resection for selected intracranial lesions. SRS combines the elements of rigid, reproducible head fixation within a stereotactic frame, precise localization of intracranial lesions with CT or MRI and precision irradiation of these targets using multiple, highly collimated small diameter radiation fields. Using this technology, discrete lesions within the brain can be irradiated safely with large, single fractions of radiation. SRS as a boost added to external beam, whole brain radiotherapy has been reported in 3 randomized controlled trials. In the trial by Kondziolka et al improved local control and overall survival was noted among a small (n=40) group of patients with 2 or 3 metastases randomized between WBXRT and WBXRT + SRS. Chougule et al randomized patients with 1-3 brain metastases to SRS alone, SRS + WBXRT or WBXRT alone. Improved local control for the radiosurgery groups and combined local and distant intracranial control for the radiosurgery + WBXRT group was noted. In the largest randomized trial of radiosurgery, the RTOG 9508 patients with 1-3 brain metastases were randomized between WBXRT and WBXRT + SRS boost.

Improvements in local control, steroid use and performance status were noted among the radiosurgery treated patients with survival improvements noted for some patient subgroups. Clearly, increased intracranial control can carry benefits to the patient in terms of a decreased rate of neurologic morbidity and death from progressive intracranial disease. However, in the setting of metastatic cancer, expected survival is limited and in the randomized trials reported, an overall survival benefit from SRS + WBXRT or Sx+ WBXRT has not uniformly been noted. From a societal point of view, it may be difficult to justify

resource intense treatments such as craniotomy or radiosurgery and from a patient point of view, it may be difficult to justify the inconvenience and side effects of these treatments in the face of a limited survival benefit. Improvements in technology to achieve improved intracranial disease control while minimizing resource use and patient inconvenience are needed for this common cancer problem.

Helical tomotherapy combines intensity modulated fan-beam radiotherapy delivery with megavoltage computed tomography imaging providing the capability for on-line image guided conformal delivery. By combining an intensity modulated helical fan beam delivery system with pre-treatment cross sectional imaging to verify patient positioning and cross sectional dose verification, helical tomotherapy could potentially replace the invasive apparatus required for conventional radiosurgery. Furthermore, the helical tomotherapy platform could potentially allow radiosurgery type boost treatments to be given synchronously with the whole brain radiotherapy component of the treatment. A fractionated synchronous boost treatment delivered with whole brain radiotherapy could:

1) Minimize discomfort and inconvenience to the patient associated with a separate SRS procedure.
2) Offer improved intracranial control due to dose escalation in the individual metastases.
3) Capitalize on the beneficial effect of tumour cell re-assortment and re-oxygenation with fractionation.
4) Minimize the potential adverse effect of tumour cell repopulation by keeping the overall treatment time short.

Thus, the combination of whole brain radiotherapy with a synchronous boost may carry significant therapeutic gain for the patient with brain metastases compared to the conventional combination of WBXRT with a separate SRS boost.

In a previously published planning study, we sought to model the feasibility of a simultaneous in field boost (SIB) to individual brain metastases during a course of whole brain radiotherapy using helical tomotherapy IMRT. Planning CT data from 14 patients with 1-3 brain metastases were used to model an intralesional SIB delivery that yielded a total intralesional dose of 60 Gy with a surrounding whole brain dose of 30 Gy (designed to be isoeffective to whole brain radiotherapy of 30 Gy with an 18 Gy/1 fraction radiosurgery boost). Accuracy of treatment of a phantom on the helical tomotherapy unit was measured. Comparisons of helical tomotherapy

delivery versus a conventional stereotactic radiotherapy technique for a particularly challenging simulated anatomy were made.

In all cases, SIB to 60Gy with whole brain radiation to 30Gy was possible while maintaining critical structures below assigned dose limits. Estimated radiation delivery time for the SIB treatment was approximately 10 minutes per fraction. Planning and treatment of the head phantom was associated with an overall accuracy of 2 mm. Comparison to conventional non-coplanar arc fractionated stereotactic radiotherapy plan demonstrated similar target coverage and improved critical tissue sparing even for a challenging anatomy with multiple lesions in the same plane as the optic apparatus. Based on this study, use of an image guided SIB using helical tomotherapy seemed feasible and a phase I trial initiated at our institution was initiated.

Our current experience with the phase I dose escalation (35, 45, 50, 55, and 60 Gy in 10 fractions) is summarized in the following table (Table 1). We have completed this trial at 60Gy in 10 fractions without any DLT events. Therefore the recommended phase II dose is considered to be 60Gy in 10 fractions

Table 1: Current Data on Phase I Dose-escalation Trial

SIB Dose level	Evaluable at 3 months	Imaging Response	Toxicity DLT
I 35 Gy	3	PR = 2<br>PD = 1	none
II 45 Gy	11	CR = 1<br>PR = 7<br>SD = 2<br>PD = 1	none
III 50Gy	10	Pr = 5<br>SD = 2<br>PD = 3	none
IV 55 Gy	3	CR = 1<br>PR = 1<br>PD = 1	none
V 60 Gy	4	PR = 1<br>SD = 1<br>PD = 2	none

2.0 Objectives

2.1 Primary Objective

The primary outcome of interest in this study is to obtain an estimate of overall survival, six month local and CNS disease control rates as measured by the RTOG brain progression criteria in order to ensure that the fractionated treatment is not substantially inferior to RTOG historical controls.

2.2 Secondary Objectives

2.21 Assessment of differences/similarities of RTOG versus RECIST versus volumetric MRI criteria within the imaging dataset created by this project

2.22 Assessment of changes in health related quality of life over time (FACT-G, FACT-Br) by assessment of domain score changes over time.

2.23 Assessment of changes in Karnofsky performance status and MMSE cognition due to protocol therapy.

2.24 Assessment of Acute and Late Toxicity due to protocol therapy.

2.25 Assessment of changes in various MRI endpoints in relation to diffusion weighted imaging and magnetic resonance spectroscopy due to protocol therapy by analysis of the imaging dataset created in conjunction with this research project.

3.0 Patient Selection

3.1 Inclusion Criteria

3.1.1 Histologic diagnosis of primary cancer

3.1.2 Contrast enhanced MRI (demonstrating 1-3 metastases within 6 weeks of enrollment

3.1.3 Age ≥ 18

3.1.4 KPS ≥ 70

3.1.5 Patient available for subsequent follow-up appointments and testing

3.1.6 Anticipated survival (independent of the brain metastases) > 3 months

3.1.7 Patient informed consent obtained

3.1.8 Extracranial disease controlled, to be treated, or absent

3.2 Exclusion Criteria

3.2.1 Metastases not suitable for synchronous boost:

3.2.1.1 > 3 lesions or any lesion size > 30 mm in maximum dimension

3.2.1.2 Metastases involving (within 5mm) brainstem or optic apparatus

3.2.1.3 Cytologic or imaging evidence of leptomeningeal spread

3.2.1.4 Intracranial extension of an osseous (calvarial) metastasis

3.2.1.5 Evidence of intraventricular or subependymal growth

3.2.2 No prior histologic confirmation of malignancy

3.2.3 Underlying medical condition precluding adequate follow-up

3.2.4 Prior cranial radiotherapy

3.2.5 Concurrent cytotoxic chemotherapy

3.2.6 Lack of informed consent

3.2.7 Unable to complete study questionnaires

3.2.8 Contraindications to MRI or gadolinium constrast

4.0 Pre-treatment Evaluations and Management

4.1 Contrast enhanced MRI brain within six weeks prior to study enrolment

4.2 Dictated history including steroid and anti-convulsant usage

4.3 Dictated physical examination

4.4 Full neurological examination with mini-mental status examination (MMSE)

4.5 FACT-Br HRQoL questionnaire during enrolment visit

4.6 Baseline NCI-CTC toxicity assessment

4.7 Concomitant medications

4.7.1 Include Corticosteroids (e.g. Decadron), Anti-epileptics (e.g. Dilantin), H_2 Blockers or Proton Pump Inhibitors (e.g. Ranitidine) as well as any systemic/anticancer treatments received by the study subjects prior to initiation and during study treatment will be recorded on the subject's case report form. Cytotoxic chemotherapy is not permitted during the radiotherapy. Patients may be treated with glucocorticoids and prophylactic anticonvulsants as required (see guidelines below).

4.7.2 Guidelines for Glucocorticoid Use

Most patients will require treatment with glucocorticoids at initial presentation. Glucocorticoids should be tapered as tolerated once treatment has commenced. Coverage is left to the discretion of the individual investigators.

4.7.3 Guidelines for Anticonvulsant Use

Patients presenting with seizures at presentation will generally require anticonvulsant therapy. For patients requiring anticonvulsants, adequate serum levels should be confirmed prior to surgery or radiosurgery and the dose adjusted accordingly. Prophylactic use of anticonvulsants is left to the discretion of the individual investigators.

5.0 Study Logistics

5.1 Pre-requisites for non-LRCP patient enrolment into trial

5.1.1 Participating centres must have Helical Tomotherapy technology available at their centre

5.1.2 Copy of local REB acceptance letter to be sent to coordinating centre PI

5.1.3 Copy of local clinical trial unit acceptance letter of trial to be sent to coordinating centre PI

5.1.4 Successfully completed dry run case (3 mets) planned with HT and subsequently confirmed by phantom delivery and measurement. Data to be submitted to coordinating centre for assessment and quality assurance approval by physics PI and nominated PI.

5.1.5 Default planning importance, penalty factors, and precedence factors to be submitted to and approved by coordinating centre PI

5.2 Database Report Form and Registration Requirements

5.2.1 Local report forms need to be approved by coordinating centre prior to patient enrolment to ensure compatibility with study database

5.2.2 Registration procedure – Please call the data co-ordinator at the LRCP to notify of potential eligibility. Eligibility requirements (first 3 pages of the on study form and signed letter of information are to be faxed to the co-ordinator at the LRCP. To complete the registration you must call the data co-ordinator immediately after faxing. If the patient is eligible the co-ordinator will confirm and provide a patient ID number

6.0 Trial Design

6.1 This clinical trial is designed to be a phase II multiinstitutional assessment of helical tomotherapy simultaneous boost radiation to 1-3 brain metastases in patients with a variety of primary malignancies

6.2 A common dose of the 60 Gy in 10 fractions will be used as a result of the results of the previous phase I dose-escalation trial coordinated by LRCP

6.3 No blinding or randomization procedures are required in this protocol. However, maximal accrual to the following four bins have been instituted in order to balance the primary site of disease as well as brain met number for an accurate historical comparison to the RTOG stereotactic trial.

6.3.1 Bin 1: Lung Primary and Solitary Met, n=33

6.3.2 Bin 2: Lung Primary and 2-3 Mets, n=19

6.3.3 Bin 3: Non-Lung Primary and Solitary Met, n=26

6.3.4 Bin 4: Non-Lung Primary and 2-3 Mets, n=15

6.4 The primary outcome of interest is the determination of overall survival Sample size for this phase II study will be based on determination of this survival rate and ensuring that it is not clinically and statistically inferior to RTOG historical controls (see section 12.0)

6.5 Other outcomes of interest include six month local and CNS disease control as measured by the RTOG brain progression criteria, RECIST criteria response, HRQoL, cognitive function, and treatment toxicity/safety

6.6 A patient will be considered evaluable for this protocol with the combination of study enrollment and initiation of protocol radiation therapy.

ENROLLMENT

Record Baseline Patient, Tumour, and Treatment Factors, FACT-Br Health-Related Quality-of-Life, MMSE, and NCI-CTC toxicity

↓

TREATMENT

Whole Brain XRT 30Gy/10 fractions with Simultaneous Infield Boost of Brain Lesions to 60Gy

↓

FOLLOW-UP

Primary Outcome: Overall survival, Local and CNS Control (RTOG criteria)

Secondary Outcomes: RECIST Criteria Response, NCI-CTC Toxicity, MMSE, and FACT-Br Health-Related Quality-of-Life

7.0 Radiation Therapy

7.1 Immobilization and Imaging

Patients will be immobilized prior to treatment in the supine position, head neutral with a custom thermoplastic s-frame shell. Axial images (3mm slice thickness, 3mm spacing) of the head to the level of C1/C2 will be acquired in the treatment position using CT simulation with contrast enhancement (if a recent contrast enhanced MRI or CT is not available for fusion for treatment planning). An isocentre located at the centre of the skull will be localized and marked on the thermoplastic shell. The treatment planning CT images will be fused with the most recent diagnostic MRI or CT.

7.2 Contouring Requirements

The following target normal structures shall be contoured on every CT study (using fusion with MRI if possible).

GTV: All brain metastasis without margin
BRAIN: Brain (R.L frontal cerebellum + temporal, parietal, occipital lobes contoured as one structure)
PTV BRAIN: Brain with 2 mm margin
BRAINSTEM: Brainstem/cord to the level of C1/C2
CHIASM: Optic chiasm
RON, LON: Optic nerves (R and L)
REYE, LEYE: Globe of eye (R and L)
RLENS, LLENS Lens of eye (R and L)
RLENSMARG, LLENSMARG: RLENS and LLENS with 5 mm margin
Note: If Brainstem or optic chiasm is very close to GTV, additional structures with 5 mm margin around these normal structures are created and used for optimization with the same constraints.

7.3 Helical Tomotherapy Treatment Planning

The CT images and contours will be exported from the CT simulator to the helical tomotherapy treatment planning system. Planning parameters to be used for helical tomotherapy treatment planning will be fan beam thickness: 5 cm; pitch = 0.215; modulation factor = 3.0; normal calculation grid (256 × 256 matrix in transversal slices). Target and normal tissue constraints to be used for planning are listed in Table 2. An example of a sample case planned for whole brain radiotherapy with SIB is illustrated in appendix 3.

7.4 Derivation of Normal Tissue Constraints and Prescription Dose

The linear quadratic equation provides a method of comparing different radiation fractionation schemes according to their Biologically Effective Dose (BED):

$$BED = nd * (1 + d/(\alpha/\beta))$$

n = number of fractions; d = dose per fraction; α/β = 10 for tumour; 3 for late effects

The LQ equation is widely accepted as a useful tool for comparing fractionation schemes and clinical trials of altered fractionation based on predictions by the model, and have confirmed its accuracy in predicting late effects between different radiation schedules. For the purposes of this study the tissue tolerances based on the BED equivalent tissue derived from available phase I/II and II trials of radiosurgery added to whole brain radiotherapy for patients with brain metastases are used for comparison (Table 3).

Within the RTOG 90-05 radiosurgery dose escalation protocol, metastasis in the 3-4cm range were treated with a radiosurgery dose of 18Gy, while smaller lesions were treated with doses up to 24Gy without toxicity. Within the RTOG 9508 trial, radiosurgery boosts of between 15-20 Gy were used in addition to a whole brain dose of 37.5Gy in 15 fractions for a total lesion BED of 159-222Gy. Using the synchronous boost technique, an intra-lesion dose of 45-60Gy/10 delivered with a whole brain dose of 30Gy/10 fractions provides similar BED range to that delivered within the RTOG 9508 (Table 2).

Podgorsak et al published a dosimetric comparison of radiosurgery techniques using high photon beams. Assuming a 1 cm lesion was to be encompassed by the 90% isodose (the prescription isodose), significant differences in the radiosurgical dose profile were noted between radiosurgical techniques at 5-15mm from the target. For instance, at 5mm from the target, dose ranged from 10% to 40% (median 25%); at 10mm < 5% to 25% (median 15%), and at 15mm < 5% to 20% (median 10%). Assuming a dose of 18Gy prescribed to the 90% isodose lines, these doses would correspond to BEDs of 13Gy, 6Gy and 3Gy at 5, 10 and 15mm from the target. Added to the BED of 69Gy (37.5Gy in 105 fractions) from the whole brain component as per the RTOG 9508, total BED to the brain surrounding the focal target of 82Gy, 75Gy and 72Gy at 5, 10 and 15mm respectively. This would correspond to doses of 37, 35 and 34 Gy when given in 10 fractions. Likewise, a maximum dose of 35Gy in 10 fractions for

brainstem and chiasm was estimated assuming a tolerance of 50Gy/25 fractions (Table 2).

7.5 Radiation Treatment Process

Following completion of the treatment planning, patients will receive their treatments on the helical tomotherapy unit as outlined below. Patients will be treated at 3Gy/day, Monday to Friday, to a total dose of 30Gy to the brain with a total SIB dose of 60 Gy to the individual metastases. Patients will be placed on the helical tomotherapy couch in the supine position with the plastic immobilization shell. The patient will be aligned to the fiducial marks on the mask with fiducial external lasers and a helical megavoltage CT scan of the head will be obtained. The daily megavoltage CT scan will be fused with the pre-treatment scan and a registration offsets in the x, y, z and rotational axis obtained. Provide a registration error of ≤ 3mm in the x,y,z directions and a rotational error ≤3° is obtained, the patient will be treated.

If registration errors greater than 3mm or 3° are obtained, the patient will be repositioned and a repeat megavoltage CT scan will be obtained. After 3 iterations, if a satisfactory patient position cannot be obtained, the treatment will be scored as a "technical failure" and they will either be treated on a conventional linear accelerator for that day or a new attempt will be performed on the next treatment day. Upon completion of the 10 fractions of radiotherapy, if > 2 fractions were scored as a "technical failure", the treatment for that patient will be scored as a "treatment failure". Modifications in the delivery technique, immobilization and repositioning techniques may be introduced for successive patients if "treatment failures" occur.

If three technical failures occur on any particular patient, a switch to a linear accelerator treatment completing the whole brain component of the radiotherapy (continuing whole brain radiotherapy at 3 Gy/day) can be exercised by the principal investigator and/or treating radiation oncologist.

TABLE 2: PLANNING DOSE CONSTRAINTS

	TYPE	IMP	DMAX Gy	DMAXPEN	DVHVOL %	DVHDOSE Gy	DVH PEN	DMIN Gy	DMINPEN
GTV	Tumour	10	60	10	95	60	n/a	60	100
PTV BRAIN	Tumour	10	60	1	95	30	n/a	30	100
RLENS	OAR	not used for optimization, but displayed							
LLENS	OAR	not used for optimization, but displayed							
RLENSMARG	OAR	3	6	1	1	5.9	3	n/a	n/a
LLENSMARG	OAR	3	6	1	1	5.9	3	n/a	n/a
REYE	OAR	1	20	1	1	19.9	1	n/a	n/a
LEYE	OAR	1	20	1	1	19.9	1	n/a	n/a
CHIASM	OAR	10	30	1	1	29	33	n/a	n/a
BRAINSTEM	OAR	10	30	1	1	29	33	n/a	n/a
RON	OAR	1	30	1	1	29	1	n/a	n/a
LON	OAR	1	30	1	1	29	1	n/a	n/a
Brain	OAR	not used for optimization, but displayed							

IMP=importance, DMAX=maximum dose, PEN=penalty, VOL=volume,
DVH=dose volume histogram, DMIN=minimum dose, OAR=organ at risk, n/a=not applicable

TABLE 3: COMPARISONS OF RADIOSURGERY FRACTIONATION SCHEMES

FRACTIONS	FRACTION SIZE	TOTAL DOSE	BED 3	BED 10	TOTAL BED 3	TOTAL BED 10*
Whole Brain (WB) Radiation Alone						
10	3	30	60	39		
15	2.5	37.5	69	47		
WB + Radiosurgery Boost						
1	15	15	90	38	159	77
1	18	18	126	50	195	97
1	20	20	153	60	222	107
1	24	24	216	82	285	128
WB with Simultaneous Infield Boost						
10	3.5	35	76	47	136	86
10	4.5	45	113	65	173	104
10	5	50	133	75	193	114
10	6	60	180	96	240	135

* BED (biological equivalent dose) including whole brain + radiosurgery components

8.0 Patient Assessments

8.1 Contrast enhanced MRI (see 8.1.4) at 3 and 6 months post-treatment.

8.1.1 Both the treating radiation oncologist and the reference radiologist will measure and calculate the bidimensional product for each of the 1-3 brain metastases identified at baseline. The bidimensional product is defined as the largest dimension multiplied by the second largest dimension that is perpendicular to it (the largest dimension). This value will be recorded on the baseline form and every subsequent follow-up form. The appearance (yes/no) of any new brain metastases and/or central/brain necrosis will be recorded on all follow-up forms. Progressive central neurological deterioration without confirmatory imaging will be coded as such for sensitivity analysis with regards to progression and toxicity. Any discrepancies between the oncologist and radiologist will be adjudicated by the local site principal investigator and/or nominated study principal investigator.

8.1.2 Definition of CNS Progression

CNS progression will be defined as a defined increase (see below) in perpendicular bi-dimensional tumor area for any of the 1-3 tracked brain metastases, or the appearance of any new brain metastasis on a follow-up scan. For lesions smaller than 1 cm in maximum diameter, a maximum increase of 50% in perpendicular bi-dimensional treatment area will be necessary to score as progression. This caveat is included to account for potential variability in measurement, which will be most susceptible to proportionate errors at smaller sizes. For greater than 1 cm lesions, the definition will use a 25% rule for change.

For progression of disease to be called, the patient must be on a stable or increasing level of steroids in order to avoid pseudoprogression of disease. Early repeat imaging (2-4 weeks) with stable steroid levels may be required in order to rule out pseudoprogression.

8.1.3 RECIST criteria progression

Brain mets response to protocol treatment will also be assessed using the RECIST criteria (Appendix V) by the treating radiation oncology at each follow-up scan.

8.1.4 Magnetic Resonance Imaging

Patients will be evaluated for objective tumor assessment by contrast enhanced MRI. MRI will be performed on high-field magnets (1.5T). The following MRI protocol is suggested: axial diffusion weighted imaging, sagittal T1W, T2W or FLAIR imaging in two planes, post gadolinium T1W sequences in axial and coronal planes (one of which must have corresponding pre-gadolinium T1W sequence). The post gadolinium images are acquired immediately following intravenous infusion of 0.1mmol/kg gadolinium.

8.2 Institutional standard of Care Brain imaging at 9, 12, 18, and 24 months by contrast enhanced CT brain or MRI brain.

8.3 Karnofsky Performance Status Assessment at 6 weeks and 3, 6, 9, 12, 18, and 24 months.

8.4 NCI-CTC Toxicity Assessment at 6 weeks and 3, 6, 9, 12, 18, and 24 months.

8.5 FACT-Br HRQoL Assessment at 6 weeks and 3, 6, 9, 12, 18, and 24 months.

8.6 Mini-mental status examination at 6 weeks and 3, 6, 9, 12, 18, and 24 months.

8.7 Assessment of Steroid and Anti-convulsant medications at 6 weeks and 3, 6, 9, 12, 18, and 24 months.

9.0 Adverse Events

9.1 Definitions

Adverse Event (AE) or reaction is any unfavorable and unintended sign (including an abnormal laboratory finding), symptom, or disease temporally associated with the use of a medical treatment or procedure that may or may not be considered related to the medical treatment or procedure.

Serious Adverse Event (SAE) or reaction as defined in the ICH Guideline: Clinical Safety Data Management: Definitions and Standards for Expedited Reporting, E2A Section IIB includes any untoward medical occurrence that at any dose:

- Results in death
- Is life-threatening (refers to an event in which the patient was at risk of death at the time of the event; it does not refer to an event which hypothetically might have caused death if it were more severe.)
- Results in persistent or significant disability/incapacity
- Requires in-patient hospitalization or prolongation of existing hospitalization
- Is a congenital anomaly/birth defect

Important medical events that may not be immediately life-threatening or result in death or hospitalization may be considered a serious adverse event, when, based upon medical and scientific judgment, they may jeopardize the patient or may require intervention to prevent one of the other outcomes listed in the definition above. Unexpected adverse reaction is one that the nature and severity is not consistent with the applicable product information (e.g., Investigator's Brochure or Product Monograph, described in the REB/IRB approved research protocol or informed consent document), or occurs with more than expected frequency.

9.2 Causality (attribution)

An adverse event or reaction is considered related to the research intervention if there is a reasonable possibility that the reaction or event may have been caused by the research intervention (i.e. a causal relationship between the reaction and the research intervention cannot be ruled out by the investigator(s)).

The relationship of an AE to the study treatment (causality) will be described using the following definitions:

Unrelated: Any adverse event for which there is evidence that an alternative etiology exists or for which no timely relationship exists to the administration of the study treatment and the adverse event does not follow any previously documented pattern. The adverse event, after careful consideration by the investigator, is clearly and incontrovertibly due to causes other than the intervention.

Unlikely: Any adverse event for which the time relationship between the study treatment and the event suggests that a causal relationship is unlikely and/or the event is more likely due to the subject's clinical condition or other therapies concomitantly administered to the subject.

Possible: Any adverse event occurring in a timely manner after the administration of the study treatment that follows a known pattern to the intervention and for which no other explanation is known. The adverse event, after careful consideration by the investigator, is considered to be unlikely related but cannot be ruled out with certainty.

Probable: Any adverse event occurring in a timely manner after the administration of the study treatment that follows a known pattern to the intervention and for which no other explanation is known. The adverse event, after careful consideration by the investigator, is believed with a high degree of certainty to be related to the intervention.

Definitely Related: Any adverse event occurring within a timely manner after administration of the study treatment that is a known sequela of the intervention and follows a previously documented pattern but for which no other explanation is known. The adverse event is believed by the investigator to be incontrovertibly related to the intervention.

9.3 Severity

The severity of adverse events will be evaluated using the Common Terminology Criteria for Adverse Events (CTCAE) v3.0 grading scale (see http://ctep.cancer.gov).

Grade 1:	Mild
Grade 2:	Moderate
Grade 3:	Severe
Grade 4:	Life-threatening or disabling
Grade 5:	Death

Note: The term "severe" is a measure of intensity: thus a severe adverse event is not necessarily serious. For example, nausea of several hours' duration may be rated as severe, but may not be clinically serious.

9.4 Immediately Reportable Adverse Events

Any grade 4 or 5 adverse reaction that is definitely, probably, or possibly the result of protocol treatment must be verbally reported to the Principal Investigator and Co-Investigators within 24 hours of discovery, and the UWO Office of Research Ethics as outlined below.

All serious, unexpected adverse events or reactions regardless of causality for subjects enrolled at the local site must be reported to the Office of Research Ethics, UWO within 7 days of discovery of the event or reaction through the Local Adverse Events Report.

NOTE: include conditions that are NOT considered a SAE in this protocol, e.g., hospitalizations for routine procedures, disease progression.

The Principal Investigator should also comply with the applicable regulatory requirement(s) related to the reporting of unexpected serious adverse drug reactions to the regulatory authority(ies).

9.5 Data Safety Monitoring Plan

9.5.1 The procedures to insure the safety of participants in this multi-centre study and the oversight of data integrity will be coordinated by the London Regional Cancer Program/London Health Sciences Centre/University of Western Ontario as the central reporting entity and a Data Safety Monitoring Committee (DSMC) composed of the PIs from each participating site will regularly review the overall progress of the study, the safety data, and the protocol-defined endpoints to determine study continuation or termination.

9.5.2 As patients are accrued at external sites, all study related data will be forwarded to LRCP on an ongoing basis as per good clinical practice. This data will be completed on clinical research forms (CRF's) provided.

9.5.3 LRCP will provide regular (monthly) updates to the participating institutions as to the number of patients accrued as well as AE/SAE events

9.5.4 If external sites encounter patients where dose limiting toxicity (defined as irreversible ≥ grade 3 CNS toxicity according to CTCAE v3.0) is experienced that is attributable (in the opinion of the local investigator), LRCP should be notified immediately of the grade and nature of the toxicity, and the study number of the patient. LRCP will report all serious, unexpected adverse events to the Office of Research Ethics, UWO (see definitions and reporting requirements). Summary reports of all grade 3 and 4 adverse events and deaths will be prepared and distributed to the sites in advance of all DSMC teleconferences.

9.5.5 The safety of the patients receiving treatment with helical tomotherapy is ensured by the rigorous quality assurance measures implemented under the supervision of the PI at each site prior to each patient's treatment (described in Treatment Process).

9.5.6 The Data Safety Monitoring Committee will conduct a review of dosimetric measures (DVH of target and normal structures) and acute toxicity data (occurring in the first 90 days) for the first 3 patients accrued at each non-LRCP site.

9.5.7 The DSMC will participate in a teleconference at least every 2 months (and as necessary) to review the overall progress of the trial in terms of treatment outcome and toxicity. Acute and late radiation-induced toxicities will be reviewed as well as disease progression for the duration of the trial.

9.5.8 PIs will be responsible for their own on-site data verification, protocol compliance and AE reporting. LRCP will periodically review random cases from external sites to ensure data quality and compliance with ICH Good Clinical Practice guidelines. Any compliance issues will be discussed with the site and reviewed by the DSMC.

10.0 Subject Discontinuation/Withdrawal

Patients can withdraw consent from further participation in the study at any time. In addition, the study nominated principal investigator can also withdraw patients with document cause (see following examples). Number of patients and reason(s) for withdrawal(s) will be listed in the final manuscript.

Examples:

1. Patient desire to be withdrawn from the study for any reason.

2. A serious local or systemic adverse event or severe toxicity attributed to the study treatment, determined by the judgment of the investigator.

3. Disease progression.

4. Intercurrent illness at the discretion of the investigator.

5. Patient unwilling or unable to keep the schedule of visits.

6. Inclusion/exclusion criteria not met at baseline or during the study.

Note: If a subject is removed from the study, the clinical and laboratory evaluations that would have been performed at the end of the study should be obtained. If a subject is removed because of intolerance to study treatment, they should remain under medical observation as long as deemed appropriate by the treating physician.

11.0 Data Collection

11.1 Enrolment Data Collection

11.1.1 Patient Related Data

Patient Age
Sex
Consult Date
Informed Consent Checklist
Baseline KPS
Baseline FACT-Br HRQoL questionnaire

11.1.2 Tumour Related Data

Primary Site
Primary Status (Treated and controlled, to be treated)
TNM Staging
Pathology Date
Extracranial Site(s) of Disease
Extracranial Status (Treated and controlled, to be treated)
Number, location and Size of Brain Mets

11.1.3 Treatment Related Data

Steroid Usage
Anti-convulsant Usage
Previous Surgery
Previous Radiation Therapy
Previous Chemotherapy
Planned Radiation Therapy (Dose, Fractions, and Start Date)

11.2 Radiation Therapy Data Collection

11.2.1 Delivered Radiation Therapy Dose, Fractions, Missed Fractions, Start and End Dates

11.2.2 Target Dosimetry for whole brain and GTV1-3 (see Appendix VII)

11.2.3 Normal Tissue Dosimetry for Optic nerves, chiasm, brainstem, orbits, and lenses (see Appendix VII)

11.2.4 Acute NCI-CTC toxicity events

11.3 Follow-up Data Collection (Table 4)

Date and status (alive with progression, alive without progression, dead)
MRI CNS progression (radiological and clinical)

Radiologic – see definition 8.1.2
Clinical – Neurological deterioration consistent with intracranial disease

CT versus MRI follow-up after 6 month time period
Local primary progression
Systemic disease progression
RECIST progression
FACT-Br questionnaires
KPS assessment
MMSE
Steroid and anti-conversant medications
NCI-CTC toxicity assessment

Table 4: Follow-up Data Collection

ASSESSMENT	PRE-RADIATION	RADIATION	6 WEEKS	3 MONTHS	6 MONTHS	9 MONTHS	12 MONTHS	18 MONTHS	24 MONTHS
Physical Exam	X		X	X	X	X	X	X	X
KPS/MMSE	X		X	X	X	X	X	X	X
NCICTC Toxicity	X	X (PAR FORMS)	X	X	X	X	X	X	X
FACT-Br	X		X	X	X	X	X	X	X
Dexamethasone (mg)	X		X	X	X	X	X	X	X
Standard of Care Brain Imaging (CT or MRI)						X	X	X	X
MRI	X			X	X				

12.0 Statistical Considerations

12.1 Sample Size Calculation

12.2.1 Descriptive statistics will be used to summarize the patient population, treatment-related toxicities, treatment dosing, causes of death, location of progression and performance status. Time to event outcomes will be estimated using the Kaplan-Meier method. Tables and plots will be used to better illustrate results. 95% confidence intervals will be constructed for outcomes of interest.

12.2.2 Efficacy outcomes considered of interest for this study are overall survival, overall time to intracranial tumour progression and local control rates. Results for patients treated with WBRT+SRS are extracted from the paper of Andrews et al, in which it was estimated that median overall survival was approximately 6.5 months, 6-month intracranial tumour progression-free rate was approximately 85% and 6-month local control rate was approximately 90%. It is hypothesized HT would be considered uninteresting for further study if median overall survival is ≤4.5 months, 6-month intracranial progression-free rate is ≤75%, OR 6-month local control rate is ≤80%.

12.2.3 To account for multiple testing, each individual test will be set at the $\alpha=0.025$ and $\beta=0.10$ level. All tests are 1-sided and calculations are performed using the on-line calculators available at www.crab.org/Calculators.asp - provided by the SouthWest Oncology Group (SWOG) Cancer Research and Biostatistics (CRaB) department. It is assumed that for this study, patients will be accrued over a 2 year purpose and there will be 6 months of follow-up after the last patient is accrued.

12.2.4 Thus, for overall survival, set H0: median OS=6.5 months versus HA: median OS=4.5 months, then 93 total patients are required. For the intracranial progression-free rate, set H0: 6-month rate=85% versus HA: 6-month rate=75%, then 71 total patients are required. For local control rate, set H0: 6-month rate=90% versus HA: 6-month rate=80%, then 53 total patients are required. Thus, we will aim to accrue a total of 93 enrolled and treated patients.

12.3 Secondary Outcomes/Analyses

12.3.1 Assessment of differences/similarities of RTOG versus RECIST versus volumetric MRI criteria

12.3.2 Assessment of changes in health related quality of life over time (FACT-G, FACT-Br)

12.3.3 Assessment of changes in Karnofsky performance status and MMSE cognition

12.3.4 Assessment of Acute and Late Toxicity

12.3.5 Assessment of changes in various MRI endpoints in relation to diffusion weighted imaging and magnetic resonance spectroscopy

13.0 Publication Policy

13.1 Primary Authorship Assignment

All site primary investigators and co-investigators listed on the clinical trial protocol title page will be co-authors in the manuscript.

13.2 Secondary Authorship Assignment

Physicians accruing to the trial will be eligible for authorship. This determination will be at the discretion of the nominated principal investigator.

REFERENCES

1. Tsao MN, Lloyd NS, Wong RK, Rakovitch E, Chow E, Laperriere N. Radiotherapeutic management of brain metastases: a systematic review and meta-analysis. Cancer Treat Rev. 2005 Jun;31(4):256-73.

2. Tsao MN, Mehta MP, Whelan TJ, Morris DE, Hayman JA, Flickinger JC, et al. The American Society for Therapeutic Radiology and Oncology (ASTRO) evidence-based review of the role of radiosurgery for malignant glioma. Int J Radiat Oncol Biol Phys. 2005 Sep 1;63(1):47-55.

3. Beavis AW. Is tomotherapy the future of IMRT? Br J Radiol. 2004 Apr;77(916):285-95.

4. Lightstone AW, Benedict SH, Bova FJ, Solberg TD, Stern RL. Intracranial stereotactic positioning systems: Report of the American Association of Physicists in Medicine Radiation Therapy Committee Task Group no. 68. Med Phys. 2005 Jul;32(7):2380-98.

5. Ma L, Xia P, Verhey LJ, Boyer AL. A Dosimetric Comparison of Fan-Beam Intensity Modulated Radiotherapy with Gamma Knife Stereotactic Radiosurgery for Treating Intermediate Intracranial Lesions. Int J Rad Oncol Biol Phys. 1999;45(5):1325-30.

6. Khoo VS, Oldham M, Adams EJ, Bedford JL, Webb S, Brada M. Comparison of intensity-modulated tomotherapy with stereotactically guided conformal radiotherapy for brain tumors. Int J Radiat Oncol Biol Phys. 1999 Sep 1;45(2):415-25.

7. Soisson ET, Tome WA, Richards GM, Mehta MP. Comparison of linac based fractionated stereotactic radiotherapy and tomotherapy treatment plans for skull-base tumors. Radiother Oncol. 2006 Mar;78(3):313-21.

8. Yartsev S, Kron T, Cozzi L, Fogliata A, Bauman G. Tomotherapy planning of small brain tumours. Radiother Oncol. 2005 Jan;74(1):49-52.

9. Shaw E, Scott C, Souhami L, Dinapoli R, Bahary J-P, Kline R, et al. Radiosurgery for the Treatment of Previously Irradiated Recurrent Primary Brain Tumors and Brain Metastases: Initial Report of Radiation Therapy Oncology Group Protocol 90-05. Int J Rad Oncol Biol Phys. 1996;34(3):647-54.

10. Andrews D, Scott CB, Sperduto PW, Flanders A, Gaspar L, Schell M, et al. Whole Brain Radiation Therapy With or Without Stereotactic Radiosurgery Boost for Patients with One to Three Brain Metastases: Phase III Results of the RTOG 9508 Randomised Trial. Lancet. 2004;363:1665.

11. Podgorsak EB, Pike GB, Olivier A, Pla M, Souhami L. Radiosurgery with high energy photon beams: a comparison among techniques. Int J Radiat Oncol Biol Phys. 1989 Mar;16(3):857-65.

12. Kron T, Grigorov G, Yu E, Wong E, Chen J, Kim B, et al. Optimisation of radiotherapy delivery and planning time for helical tomotherapy by means of pre-calculated planning parameters. Med Phys. 2003;30:1938.

13. Amendola BE, Wolf AL, Coy SR, Amendola M, Bloch L. Gamma knife radiosurgery in the treatment of patients with single and multiple brain metastases from carcinoma of the breast. Cancer J. 2000 Mar-Apr;6(2):88-92.

14. Kondziolka D, Patel A, Lunsford LD, Kassam A, Flickinger JC. Stereotactic Radiosurgery Plus Whole Brain Radiotherapy Versus Radiotherapy Alone for Patients with Multiple Brain Metastases. Int J Rad Oncol Biol Phys. 1999;45(2):427-34.

15. Hall EJ, DJ B. The Radiobiology of Radiosurgery: Rationale for Different Treatment Regimes for AVMs and Malignancies. IJROBP. 1993;25(2):381-5.

16. Mackie TR, Balog J, Ruchala K, Shepard D, Aldridge S, Fitchard E, et al. Tomotherapy. Semin Radiat Oncol. 1999 Jan;9(1):108-17.

17. Chase D, Ramsey C, Maha S. The Impact of Inter- and Intra-Fraction Motion in Image Guided Helical Tomotherapy. Int J Rad Oncol Biol Phys. 2004;60(1S):S619 abstract 2465.

18. Noel G, Simon JM, Valery CA, Cornu P, Boisserie G, Hasboun D, et al. Radiosurgery for brain metastasis: impact of CTV on local control. Radiother Oncol. 2003 Jul;68(1):15-21.

19. Salter BJ. NOMOS Peacock IMRT utilizing the Beak post collimation device. Med Dosim. 2001 Spring;26(1):37-45.

20. Gladwish A, Kron T, McNiven A, Bauman G, Van Dyk J. Asymmetric fan beams (AFB) for improvement of the craniocaudal dose distribution in helical tomotherapy delivery. Med Phys. 2004 Sep;31(9):2443-8.

21. Bolsi A, Fogliata A, Cozzi L. Radiotherapy of small intracranial tumours with different advanced techniques using photon and proton beams: a treatment planning study. Radiother Oncol. 2003 Jul;68(1):1-14.

22. Dogan N, King S, Emami B, Mohideen N, Mirkovic N, Leybovich LB, et al. Assessment of different IMRT boost delivery methods on target coverage and normal-tissue sparing. Int J Radiat Oncol Biol Phys. 2003 Dec 1;57(5):1480-91.

23. Khuntia D, Brown P, Li J, Mehta MP. Whole-brain radiotherapy in the management of brain metastasis. J Clin Oncol. 2006 Mar 10;24(8):1295-304.

24. Bauman G, Yartsev S, Fisher B, Kron T, Laperriere N, Heydarian M, VanDyk J. Simultaneous Infield Boost With Helical Tomotherapy for Patients With 1 to 3 Brain Metastases. American Journal of Clinical Oncology 2007 Feb;30(1):38-44.

APPENDIX I LETTER OF INFORMATION AND CONSENT FORM

COMBINED LETTER OF INFORMATION AND CONSENT FORM

A Phase II Multi-institutional Study Assessing Simultaneous In-field Boost Helical Tomotherapy for 1-3 Brain Metastases

Principal Study Investigator:

Dr. George Rodrigues, MD, FRCPC, MSc
Departments of Radiation Oncology and Epidemiology/Biostatistics
London Regional Cancer Program
London Health Sciences Centre
790 Commissioners Road E
London, ON N6A 4L6
519-685-8500 x52833

Introduction and Purpose of Study:

You are being invited to participate in a research study looking at the radiation treatment of metastatic brain cancer. The purpose of this letter is to provide information with regards to this study to allow you to make an informed decision on participating in this research. It is important for you to understand why this study is being conducted and what it will involve. Please take the time to read this carefully and feel free to ask questions to the study investigator, your doctor, or the research assistant if anything is unclear. This combined letter of information should be read by the patient who has just been seen at the London Regional Cancer Program for consultation in relation to metastatic cancer to the brain.

Background Study Information:

You have been diagnosed with an incurable cancer that has spread to the brain (brain metastases) and your physician has recommended radiation treatments to help reduce the symptoms (such as headache, seizures, or loss of function) the cancer may be causing you and to help prevent these symptoms in the future. You are being offered the opportunity to participate in a research study examining the use of a new radiation treatment machine called "Helical Tomotherapy" in the treatment of patients with cancer that has spread (metastasized) to the brain.

Helical tomotherapy (HT) is a novel radiation treatment machine that combines two existing technologies: spiral (rotating) radiotherapy treatments combined

with simultaneous computed tomography (CT) imaging of the body. This new machine can potentially allow radiation treatments to be focused more precisely, and delivered more accurately than with existing radiation machines. In this study, HT will be used to provide radiation treatments (whole brain radiotherapy, daily over 10 treatments) that are commonly used to treat cancer metastatic to the brain. In addition, the individual spots of cancer (metastases) in the brain will be treated to a higher dose (approximately 2 times higher) than the dose to the whole brain. The purpose of this study is to determine the effectiveness of whole brain radiation with lesion boosting with the HT machine.

Research Question:

This research will evaluate the effectiveness and toxicity of radiation therapy given to the metastatic cancer to the brain (1-3 lesions ≤ 3cm each). This clinical study will assess a dose level (60 Gy in 10 treatments) of radiation therapy given by an image-guided intensity modulated radiation therapy machine called Helical Tomotherapy. The total number of treated participants in the study will be 93 patients.

Study Population:

Healthy adult patients who are being seen for a consultation for metastatic cancer to the brain with 1-3 lesions on baseline MRI scanning are invited to participate in this study. Patients with poor survival characteristics, contraindications to high dose brain radiation, previous brain radiation, inability to comply with study procedures and questionnaires, or are to receive concurrent chemotherapy are ineligible to participate in the study.

Study Procedures:

If you choose to participate in this study, you will initially undergo treatment planning. The planning process will involve construction of a plastic mask to hold your head still for treatment followed by a CT scan. As part of the CT scan you may have an intravenous line placed and contrast will be injected into your vein to make the brain tumours easier to see on the scan. This will take approximately 45 minutes. The information from the CT scan will be used to target the tumour for treatment planning.

Treatments on the helical tomotherapy unit will be given daily Monday – Friday, over 2 weeks (10 treatments), using the same dose per day. A CT scan through the region being treated will be taken on the helical tomotherapy unit prior to treatment each day and your position for the treatment adjusted if necessary. Once your positioning is confirmed, the treatment will be given. If treatment cannot be delivered on the helical tomotherapy unit on a given day due to

technical difficulties (i.e. you cannot be positioned properly for treatment or the machine is not operational), your treatment for that day will be rescheduled for the following day.

You will be monitored regularly by your study doctor during the 2 weeks of treatment and regularly thereafter. A follow-up visit without scanning will be performed at 6 weeks after treatment to assess how you are doing after the treatment. Follow-up MRI scans with check-ups will be performed at 3 and 6 months after your treatment. Other brain scans and check-ups will be done at regular intervals up to 2 years after study enrollment to measure the effect of the treatment on the cancer and any side effects/quality-of-life effects of the treatment.

Research Methods

Since this is a radiation evaluation trial (otherwise known as a phase II study), all patients will receive radiation treatment at the same dose level. There is no random assignment of treatment. Side effects, tumor response, and health-related quality of life will be assessed at regular intervals. The primary outcome of interest in this study is local control of brain metastatic disease. Secondary outcomes to be assessed include survival, quality-of-life, cognitive function, and treatment toxicity.

Treatment Benefits

Potential benefits of participating in the study include the possible decrease of side effects or improved chances of controlling brain tumour metastases using the high-dose radiation therapy utilizing the helical tomotherapy unit.

Treatment Risks

The placement of the intravenous line for the planning CT scan will be associated with the discomfort similar to an injection or drawing blood by a needle. The injection of the intravenous contrast for the scan will produce a sensation of cold and may produce an odd taste in your mouth. Rarely, an individual may have a severe allergic reaction (such as rash or trouble breathing) from the intravenous contrast that may require treatment with medications and, very rarely, may be severe enough to cause death.

Radiation treatments to treat brain metastases are typically given as daily treatments, Monday to Friday over 2 weeks (10 treatments). Side effects from this treatment depend on the area being treated.

- Radiation treatments to the head may commonly cause headache, hair loss, and mild sunburn of the skin, decreased hearing or irritation of the ears, dryness or irritation of the eyes and dry or sore mouth or throat or loss of taste during radiation treatments. These side effects usually clear up within 4-6 weeks after radiation. Hair may take up to 6 months to return or may be permanent.

- Rarely, high dose radiation to the brain may produce injury to brain tissue (necrosis) that may create swelling and worsen symptoms from the cancer in the brain or produce new symptoms (such as headaches, seizures or loss of function). If necrosis occurs, treatment with anti-swelling drugs (steroids) or surgery may be required to relieve symptoms. The higher dose of radiation being given to the individual metastases in the brain may increase the risk of necrosis occurring compared to conventional radiotherapy without boost treatment. In addition, radiation to the whole brain may cause changes to thinking processes or memory that may occur months to years after radiation.

- Fatigue during and following radiation treatments is common.

As part of your pre-treatment workup and post treatment follow-up you will be asked to have contrast enhanced MRI scans of your brain. During the MRI, you must lie still in the magnet, which is a confined space. You may get a feeling of claustrophobia (a fear of being in a narrow area or a feeling of anxiety), nervousness, or sweating. The MRI machine makes banging and humming noises. You will be provided with earplugs to reduce the noise. You will be observed by a technologist during the entire procedure and may be spoken to through an intercom in the scanner.

The Food & Drug Administration (USA) has indicated that for clinical diagnosis an 'insignificant' risk is associated with human MRI exposure at the intensities used in this project. Current Canadian guidelines follow the USA guidelines. Although very rare, injury and deaths have occurred in MRI units from unsecured metal objects being drawn at high speeds into the magnet or from internal body metal fragments of which the subject was unaware or had not informed MRI staff. To minimize this latter possibility, it is essential that you complete a screening questionnaire prior to obtaining your MRI scan. Other remote but potential risks involve tissue burns and temporary hearing loss from the loud noise inside the magnet. The latter can be avoided with ear protection that also allows continuous communication between the subject and staff during the study. There is a possibility that you will experience a localized twitching sensation due to the magnetic field changes during the scan. This is not unexpected and should not be painful. However, you can discontinue the exam at any time.

Side effects from the Gadolinium contrast agent can include mild headache, nausea, and local injection site pain. Low blood pressure and lightheadedness occur rarely (<1%). Allergy to Gadolinium is very rare (less than 1 in one thousand patients) which can manifest itself as hives, itchy eyes, and shortness of breath.

Privacy and Confidentiality:

All data that will be collected from this study will be considered confidential. We will maintain your confidentiality by using a unique identifier number on all documents instead of your name. A separate secure document will contain the linkage between your name and identifier number in order to minimize the possibility of a breach of you privacy. Your research records will be stored in a locked cabinet at the clinical trials unit. Once the data has been put into the research database, any identifying information will be removed from the database in order to protect your confidentially. If the results of the study are published, your name will not be used and no information that discloses your identity will be released or published without your explicit consent. By signing this consent form; you hereby consent to participation in this study. By consenting to this study you agree to allow us to confidentially collect this data. If you do not consent to this data collection, then you cannot participate in this study. Representatives of The University of Western Ontario Health Sciences Research Ethics Board and the research team at your hospital may contact you or require access to your study-related records to monitor the conduct of the research.

If, during the course of this study, new information becomes available that may relate to your willingness to continue to participate, this information will be provided to you by the investigator.

Patient Rights:

Your participation in this study is voluntary. You may refuse to participate, refuse to answer any questions, or you may withdraw from the study at any time with no affect on your future care. If you decide not to participate or if you withdraw from the study before it is completed, the alternative procedures or courses of action will be explained to you by your doctor. A Data Safety Monitoring Committee will be reviewing the data from this research on a regular basis throughout the study. This will ensure that the participants are not exposed to increased risks as part of the study. If you are already participating in another study at this time, please inform the study doctor right away to determine if it is appropriate for you to participate in this study. We will tell you about new information that may affect your health, welfare, or willingness to stay in this

study. If the results of the study are published, your name will not be used. If you would like to receive a copy of the overall results of this study, please put your name and address on a blank piece of paper and give it to the Clinical Research Associate.

If you have any questions about your rights as a research participant or the conduct of the study you may contact VP Research, Chief Administrator Officer, Lawson Health Research Institute, 519-667-6649.

Compensation and Costs:

There is no compensation to you in relation to this research study. A copy of this letter is for you to keep. Taking part in the study may result in added costs to you (e.g. parking, travel to the cancer clinic, etc.). In the event you are injured as a consequence of participation in this study due to the administration of the study treatment and/or procedure(s), your medical condition will be evaluated and medical care will be provided by one of the investigators or you will be referred for appropriate treatment. Although no funds have been set aside to compensate you in the event of injury or illness related to the study treatment or procedures, you do no waive any or your legal rights for compensation by signing the consent form.

Consent Statement:

I have read the Combined Letter of Information and Consent Form, have had the nature of the study explained to me and I agree to participate. All questions have been answered to my satisfaction.

______________	______________	______________
Patient Name	Patient Signature	Date

______________	______________
Person Conducting Informed Consent Discussion (Print)	Person Conducting Informed Consent Discussion (Sign)

APPENDIX II PERFORMANCE STATUS SCORING

Karnofsky Performance Status Scale

PERFORMANCE STATUS	DESCRIPTION
100	No complaints, no evidence of disease
90	Able to carry on normal activity, minor signs or symptoms of disease.
80	Some signs or symptoms of disease, carries on normal activity with some effort.
70	Cares for self, unable to carry on normal activity or to do active work.
60	Requires occasional assistance but is able to care for most personal needs.
50	Requires considerable assistance and frequent medical care.
40	Disabled, requires special care and assistance
30	Severely disabled, hospitalization indicated although death not imminent.
20	Very sick; hospitalization necessary, requires active supportive treatment.
10	Moribund; fatal processes progressing rapidly
0	Dead

APPENDIX III SAMPLE SIB HELICAL TOMOTHERAPY PLAN

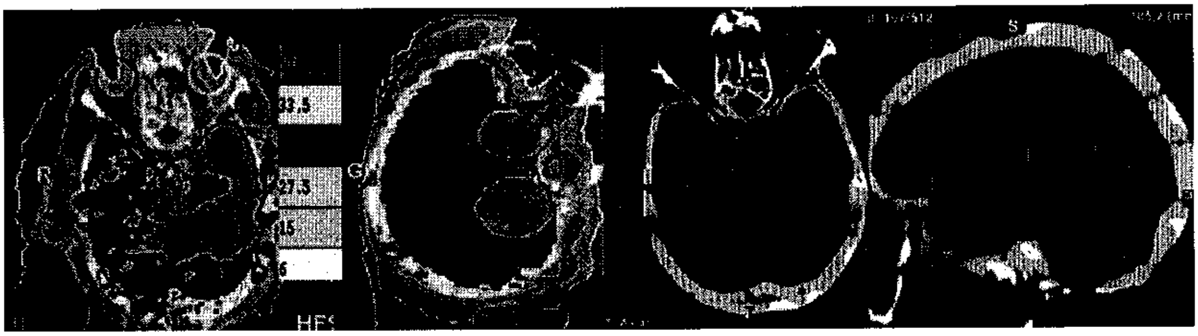

FIGURE 1. Helical tomotherapy for a phantom case with 3 lesions where 2 lesions are located in the same plane. Despite the coplanar delivery inherent in helical tomotherapy, good sparing of brainstem, optic chiasm, and eyes is maintained.

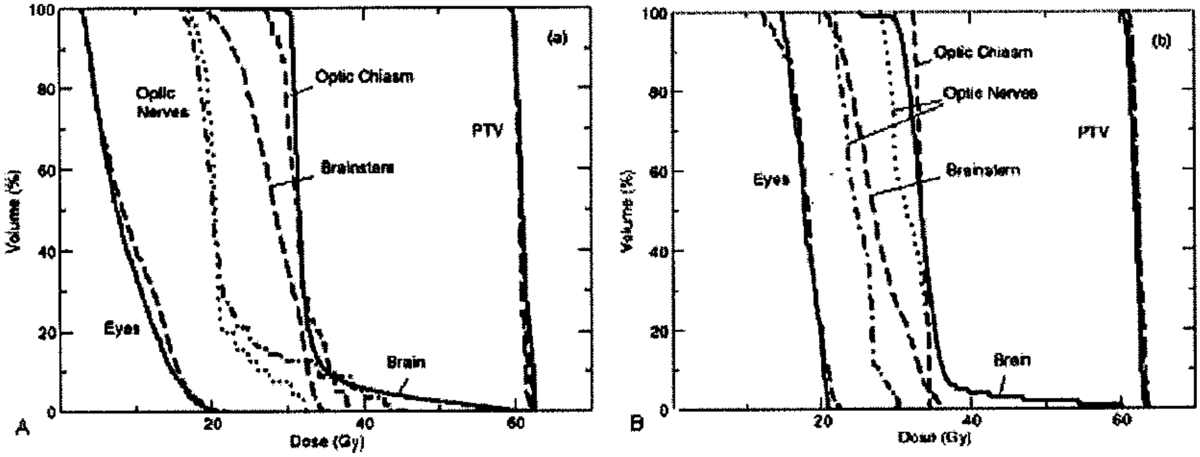

FIGURE 2. Dose volume histograms for tomotherapy SIB plan (A) and a conventional radiosurgery plan (B) for the same case.

APPENDIX IV RECIST CRITERIA

Response Evaluation Criteria in Solid Tumors (RECIST) Quick Reference:

Eligibility

- Only patients with measurable disease at baseline should be included in protocols where objective tumor response is the primary endpoint.

  Measurable disease - the presence of at least one measurable lesion. If the measurable disease is restricted to a solitary lesion, its neoplastic nature should be confirmed by cytology/histology.

  Measurable lesions - lesions that can be accurately measured in at least one dimension with longest diameter ≥20 mm using conventional techniques or ≥10 mm with spiral CT scan.

  Non-measurable lesions - all other lesions, including small lesions (longest diameter <20 mm with conventional techniques or <10 mm with spiral CT scan), i.e., bone lesions, leptomeningeal disease, ascites, pleural/pericardial effusion, inflammatory breast disease, lymphangitis cutis/pulmonis, cystic lesions, and also abdominal masses that are not confirmed and followed by imaging techniques; and.

- All measurements should be taken and recorded in metric notation, using a ruler or calipers. All baseline evaluations should be performed as closely as possible to the beginning of treatment and never more than 4 weeks before the beginning of the treatment.

- The same method of assessment and the same technique should be used to characterize each identified and reported lesion at baseline and during follow-up.

- Clinical lesions will only be considered measurable when they are superficial (e.g., skin nodules and palpable lymph nodes). For the case of skin lesions, documentation by color photography, including a ruler to estimate the size of the lesion, is recommended.

Methods of Measurement –

- CT and MRI are the best currently available and reproducible methods to measure target lesions selected for response assessment. Conventional CT and MRI should be performed with cuts of 10 mm or less in slice thickness contiguously. Spiral CT should be performed using a 5 mm contiguous reconstruction algorithm. This applies to tumors of the chest, abdomen and

pelvis. Head and neck tumors and those of extremities usually require specific protocols.

- Lesions on chest X-ray are acceptable as measurable lesions when they are clearly defined and surrounded by aerated lung. However, CT is preferable.

- When the primary endpoint of the study is objective response evaluation, ultrasound (US) should not be used to measure tumor lesions. It is, however, a possible alternative to clinical measurements of superficial palpable lymph nodes, subcutaneous lesions and thyroid nodules. US might also be useful to confirm the complete disappearance of superficial lesions usually assessed by clinical examination.

- The utilization of endoscopy and laparoscopy for objective tumor evaluation has not yet been fully and widely validated. Their uses in this specific context require sophisticated equipment and a high level of expertise that may only be available in some centers. Therefore, the utilization of such techniques for objective tumor response should be restricted to validation purposes in specialized centers. However, such techniques can be useful in confirming complete pathological response when biopsies are obtained.

- Tumor markers alone cannot be used to assess response. If markers are initially above the upper normal limit, they must normalize for a patient to be considered in complete clinical response when all lesions have disappeared.

- Cytology and histology can be used to differentiate between PR and CR in rare cases (e.g., after treatment to differentiate between residual benign lesions and residual malignant lesions in tumor types such as germ cell tumors).

Baseline documentation of "Target" and "Non-Target" lesions

- All measurable lesions up to a maximum of five lesions per organ and 10 lesions in total, representative of all involved organs should be identified as ***target lesions*** and recorded and measured at baseline.

- Target lesions should be selected on the basis of their size (lesions with the longest diameter) and their suitability for accurate repeated measurements (either by imaging techniques or clinically).

- A sum of the longest diameter (LD) for *all target lesions* will be calculated and reported as the baseline sum LD. The baseline sum LD will be used as reference by which to characterize the objective tumor.

- All other lesions (or sites of disease) should be identified as ***non-target lesions*** and should also be recorded at baseline. Measurements of these lesions are not required, but the presence or absence of each should be noted throughout follow-up.

Response Criteria

	**Evaluation of target lesions**
* Complete Response (CR):	Disappearance of all target lesions
* Partial Response (PR):	At least a 30% decrease in the sum of the LD of target lesions, taking as reference the baseline sum LD
* Progressive Disease (PD):	At least a 20% increase in the sum of the LD of target lesions, taking as reference the smallest sum LD recorded since the treatment started or the appearance of one or more new lesions
* Stable Disease (SD):	Neither sufficient shrinkage to qualify for PR nor sufficient increase to qualify for PD, taking as reference the smallest sum LD since the treatment started
	**Evaluation of non-target lesions**
* Complete Response (CR):	Disappearance of all non-target lesions and normalization of tumor marker level
* Incomplete Response/ Stable Disease (SD):	Persistence of one or more non-target lesion(s) or/and maintenance of tumor marker level above the normal limits
* Progressive Disease (PD):	Appearance of one or more new lesions and/or unequivocal progression of existing non-target lesions (1)

(1) Although a clear progression of "non target" lesions only is exceptional, in such circumstances, the opinion of the treating physician should prevail and the progression status should be confirmed later on by the review panel (or study chair).

Evaluation of best overall response

The best overall response is the best response recorded from the start of the treatment until disease progression/recurrence (taking as reference for PD the smallest measurements recorded since the treatment started). In general, the patient's best response assignment will depend on the achievement of both measurement and confirmation criteria

Target lesions	Non-Target lesions	New Lesions	Overall response
CR	CR	No	CR
CR	Incomplete response/SD	No	PR
PR	Non-PD	No	PR
SD	Non-PD	No	SD
PD	Any	Yes or No	PD
Any	PD	Yes or No	PD
Any	Any	Yes	PD

- Patients with a global deterioration of health status requiring discontinuation of treatment without objective evidence of disease progression at that time should be classified as having "symptomatic deterioration". Every effort should be made to document the objective progression even after discontinuation of treatment.
- In some circumstances it may be difficult to distinguish residual disease from normal tissue. When the evaluation of complete response depends on this determination, it is recommended that the residual lesion be investigated (fine needle aspirate/biopsy) to confirm the complete response status.

Confirmation

- The main goal of confirmation of objective response is to avoid overestimating the response rate observed. In cases where confirmation of response is not feasible, it should be made clear when reporting the outcome of such studies that the responses are not confirmed.

- To be assigned a status of PR or CR, changes in tumor measurements must be confirmed by repeat assessments that should be performed no less than 4 weeks after the criteria for response are first met. Longer intervals as determined by the study protocol may also be appropriate.
- In the case of SD, follow-up measurements must have met the SD criteria at least once after study entry at a minimum interval (in general, not less than 6-8 weeks) that is defined in the study protocol

Duration of overall response

- The duration of overall response is measured from the time measurement criteria are met for CR or PR (whichever status is recorded first) until the first date that recurrence or PD is objectively documented, taking as reference for PD the smallest measurements recorded since the treatment started.

Duration of stable disease

- SD is measured from the start of the treatment until the criteria for disease progression are met, taking as reference the smallest measurements recorded since the treatment started.
- The clinical relevance of the duration of SD varies for different tumor types and grades. Therefore, it is highly recommended that the protocol specify the minimal time interval required between two measurements for determination of SD. This time interval should take into account the expected clinical benefit that such a status may bring to the population under study.

Response review

- For trials where the response rate is the primary endpoint it is strongly recommended that all responses be reviewed by an expert(s) independent of the study at the study's completion. Simultaneous review of the patients' files and radiological images is the best approach.

Reporting of results

- All patients included in the study must be assessed for response to treatment, even if there are major protocol treatment deviations or if they are ineligible. Each patient will be assigned one of the following categories: 1) complete response, 2) partial response, 3) stable disease, 4) progressive disease, 5) early death from malignant disease, 6) early death from toxicity, 7) early death because of other cause, or 9) unknown (not assessable, insufficient data).

- All of the patients who met the eligibility criteria should be included in the main analysis of the response rate. Patients in response categories 4-9 should be considered as failing to respond to treatment (disease progression). Thus, an incorrect treatment schedule or drug administration does not result in exclusion from the analysis of the response rate. Precise definitions for categories 4-9 will be protocol specific.

- All conclusions should be based on all eligible patients.

- Subanalyses may then be performed on the basis of a subset of patients, excluding those for whom major protocol deviations have been identified (e.g., early death due to other reasons, early discontinuation of treatment, major protocol violations, etc.). However, these subanalyses may not serve as the basis for drawing conclusions concerning treatment efficacy, and the reasons for excluding patients from the analysis should be clearly reported.

- The 95% confidence intervals should be provided.

Patient ID#:________ Date:__-___-__
yy mmm dd

Appendix V: Protocol: Tomo SIB Phase II Patient Initials:________

FACT-Br (Version 4)

Below is a list of statements that other people with your illness have said are important. **By circling one (1) number per line, please indicate how true each statement has been for you <u>during the past 7 days.</u>**

	PHYSICAL WELL-BEING	Not at all	A little bit	Some-what	Quite a bit	Very much
GP1	I have a lack of energy	0	1	2	3	4
GP2	I have nausea	0	1	2	3	4
GP3	Because of my physical condition, I have trouble meeting the needs of my family	0	1	2	3	4
GP4	I have pain	0	1	2	3	4
GP5	I am bothered by side effects of treatment	0	1	2	3	4
GP6	I feel ill	0	1	2	3	4
GP7	I am forced to spend time in bed	0	1	2	3	4

	SOCIAL/FAMILY WELL-BEING	Not at all	A little bit	Some-what	Quite a bit	Very much
GS1	I feel close to my friends	0	1	2	3	4
GS2	I get emotional support from my family	0	1	2	3	4
GS3	I get support from my friends	0	1	2	3	4
GS4	My family has accepted my illness	0	1	2	3	4
GS5	I am satisfied with family communication about my illness	0	1	2	3	4
GS6	I feel close to my partner (or the person who is my main support)	0	1	2	3	4
Q1	*Regardless of your current level of sexual activity, please answer the following question. If you prefer not to answer it, please check this box* ☐ *and go to the next section.*					
GS7	I am satisfied with my sex life	0	1	2	3	4

Copyright 1987, 1997

Patient Initials:________ Patient ID#:__________ Date:__-___-__
yy mmm dd

FACT-Br (Version 4)

By circling one (1) number per line, please indicate how true each statement has been for you <u>during the past 7 days.</u>

	EMOTIONAL WELL-BEING	Not at all	A little bit	Some-what	Quite a bit	Very much
GE1	I feel sad	0	1	2	3	4
GE2	I am satisfied with how I am coping with my illness	0	1	2	3	4
GE3	I am losing hope in the fight against my illness	0	1	2	3	4
GE4	I feel nervous	0	1	2	3	4
GE5	I worry about dying	0	1	2	3	4
GE6	I worry that my condition will get worse	0	1	2	3	4

	FUNCTIONAL WELL-BEING	Not at all	A little bit	Some-what	Quite a bit	Very much
GF1	I am able to work (include work at home)	0	1	2	3	4
GF2	My work (include work at home) is fulfilling	0	1	2	3	4
GF3	I am able to enjoy life	0	1	2	3	4
GF4	I have accepted my illness	0	1	2	3	4
GF5	I am sleeping well	0	1	2	3	4
GF6	I am enjoying the things I usually do for fun	0	1	2	3	4
GF7	I am content with the quality of my life right now	0	1	2	3	4

Copyright 1987, 1997

FACT-Br (Version 4)

By circling one (1) number per line, please indicate how true each statement has been for you <u>during the past 7 days.</u>

	**ADDITIONAL CONCERNS**	**Not at all**	**A little bit**	**Some-what**	**Quite a bit**	**Very much**
Br1	I am able to concentrate	0	1	2	3	4
Br2	I have had seizures (convulsions)	0	1	2	3	4
Br3	I can remember new things	0	1	2	3	4
Br4	I get frustrated that I cannot do things I used to	0	1	2	3	4
Br5	I am afraid of having a seizure (convulsion)	0	1	2	3	4
Br6	I have trouble with my eyesight	0	1	2	3	4
Br7	I feel independent	0	1	2	3	4
NTX 6	I have trouble hearing	0	1	2	3	4
Br8	I am able to find the right word(s) to say what I mean	0	1	2	3	4
Br9	I have difficulty expressing my thoughts	0	1	2	3	4
Br10	I am bothered by the change in my personality	0	1	2	3	4
Br11	I am able to make decisions and take responsibility	0	1	2	3	4
Br12	I am bothered by the drop in my contribution to the family	0	1	2	3	4
Br13	I am able to put my thoughts together	0	1	2	3	4
Br14	I need help in caring for myself (bathing, dressing, eating, etc.)	0	1	2	3	4
Br15	I am able to put my thoughts into action	0	1	2	3	4
Br16	I am able to read like I used to	0	1	2	3	4
Br17	I am able to write like I used to	0	1	2	3	4
Br18	I am able to drive a vehicle (my car, truck, etc.)	0	1	2	3	4
Br19	I have trouble feeling sensations in my arms, hands, or legs	0	1	2	3	4
Br20	I have weakness in my arms or legs	0	1	2	3	4
Br21	I have trouble with coordination	0	1	2	3	4
An 10	I get headaches	0	1	2	3	4

US English
Copyright 1987, 1997

The Mini-Mental State Exam

Patient________________________________ Examiner ______________________ Date__________

Maximum	Score	
		**Orientation**
5	( )	What is the (year) (season) (date) (day) (month)?
5	( )	Where are we (state) (country) (town) (hospital) (floor)?
		**Registration**
3	( )	Name 3 objects: 1 second to say each. Then ask the patient all 3 after you have said them. Give 1 point for each correct answer. Then repeat them until he/she learns all 3. Count trials and record. Trials __________
		**Attention and Calculation**
5	( )	Serial 7's. 1 point for each correct answer. Stop after 5 answers. Alternatively spell "world" backward.
		**Recall**
3	( )	Ask for the 3 objects repeated above. Give 1 point for each correct answer.
		**Language**
2	( )	Name a pencil and watch.
1	( )	Repeat the following "No ifs, ands, or buts"
3	( )	Follow a 3-stage command: "Take a paper in your hand, fold it in half, and put it on the floor."
1	( )	Read and obey the following: CLOSE YOUR EYES
1	( )	Write a sentence.
1	( )	Copy the design shown.

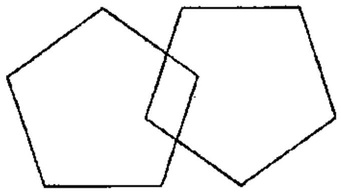

_____ Total Score

ASSESS level of consciousness along a continuum __________

Alert Drowsy Stupor Coma
